# Challenges of Investigating Compartmentalized Brain Energy Metabolism Using Nuclear Magnetic Resonance Spectroscopy in vivo

**DOI:** 10.1007/s11064-024-04324-4

**Published:** 2025-01-04

**Authors:** João M. N. Duarte

**Affiliations:** 1 Department of Experimental Medical Science, Faculty of Medicine, Lund University, Lund, Sweden; 2 Wallenberg Centre for Molecular Medicine, Lund University, Lund, Sweden

**Keywords:** Energy metabolism, Neurochemicals, Glutamate, GABA, Magnetic resonance

## Abstract

Brain function requires continuous energy supply. Thus, unraveling brain metabolic regulation is critical not only for our basic understanding of overall brain function, but also for the cellular basis of functional neuroimaging techniques. While it is known that brain energy metabolism is exquisitely compartmentalized between astrocytes and neurons, the metabolic and neuro-energetic basis of brain activity is far from fully understood. ^1^H nuclear magnetic resonance (NMR) spectroscopy has been widely used to detect variations in metabolite levels, including glutamate and GABA, while ^13^C NMR spectroscopy has been employed to study metabolic compartmentation and to determine metabolic rates coupled brain activity, focusing mainly on the component corresponding to excitatory glutamatergic neurotransmission. The rates of oxidative metabolism in neurons and astrocytes are both associated with the rate of the glutamate-glutamine cycle between neurons and astrocytes. However, any possible correlation between energy metabolism pathways and the inhibitory GABAergic neurotransmission rate in the living brain remains to be experimentally demonstrated. That is due to low GABA levels, and the consequent challenge of determining GABAergic rates in a non-invasive manner. This brief review surveys the state-of-the-art analyses of energy metabolism in neurons and astrocytes contributing to glutamate and GABA synthesis using ^13^C NMR spectroscopy in vivo, and identifies limitations that need to be overcome in future studies.

**Supplementary Information:**

The online version contains supplementary material available at 10.1007/s11064-024-04324-4.

## Introduction

Glutamate and γ-aminobutyrate (GABA) are, respectively, the major excitatory and inhibitory neurotransmitters in the central nervous system. Astrocytes have processes surrounding synapses and are, thus, perfectly poised to regulate synaptic activity, and to help taking up glutamate and GABA from the synaptic cleft following neuronal release [1]. Astrocytes oxidize glutamate and GABA [2], and synthesize glutamine that is provided to neurons, and this feature is of paramount imporance for the homeostasis of synaptic glutamate and GABA: astrocytic mitochondial metabolism and glutamine synthesis are nececssary for the development and maintenance of functional synapses [3, 4]; and deficient glutamine synthesis hampers neuronal neurotransmitter availability in animal models of neurodegeneration (e.g., [5]).

The energetic cost of brain activity, and neurotransmission in particular, is thus shared between glutamatergic neurons, GABAergic neurons and astrocytes, but the compartmentation of brain energy metabolism across activity states is not fully understood. While NMR spectroscopy coupled with ^13^C tracing has been employed to study brain energy metabolism in a non-invasive manner, there are physical and experimental limitations that render such studies difficult.

While this review focuses on the investigation of metabolism in neurons and astrocytes, it is noteworthy that metabolism in microglia and oligodendrocytes also responds to extracellular signals. Namely, metabolite shuttling between oligodendrocytes and myelinated neurons was shown to be regulated by axonal activity (e.g., [6, 7]), and microglia shift from oxidative to more glycolytic energy production when stimulated by infection or deleterious signals (e.g., [8, 9]). However, metabolic fluxes in glial cells other than astrocytes are thought to provide a relatively small contribution to brain energy metabolism supporting the glutamatergic and GABAergic neurotransmission, and they are not possible to dissect using NMR spectroscopy in vivo. Hereafter, glial energy metabolism investigated by NMR spectroscopy is considered to represent the astrocytic compartment.

### Current Knowledge of Brain Energy Metabolism

At rest, the cerebral metabolic rate of glucose (CMR_glc_) is coupled to that of O_2_ (CMR_O2_) and cerebral blood flow (CBF), such that glucose is almost completely oxidized to CO_2_ and water [10]. This appears to not be the case at high neuronal activity rates. Landmark studies found increased CMR_O2_ (6%) to be smaller than CBF and CMR_glc_ elevation (40–55%) during visual and somatosensory stimulation, suggesting that the CMR_glc_-CMR_O2_ coupling is lost in the activated state [11, 12]. Knowledge on CMR_O2_ and CBF regulation, namely including that acquired through positron emission tomography (PET) experiments, was significant for understanding the physiology behind the blood oxygen dependent (BOLD) contrast in brain functional magnetic resonance imaging (fMRI) [13]. Recently, the uncoupling between CBF and CMR_O2_ during brain activation has been proposed to be necessary for maintaining homeostasis of pH, pCO2, and pO2 upon increased glycolytic proton production [14]. In turn, the uncoupling between CMR_glc_ and CMR_O2_ implies inefficient ATP generation by glycolysis while the brain has a high aerobic capacity, and results in lactate production during intense activity that is diffused, used as substrate and released from the brain [15]. The first functional studies using ^1^H magnetic resonance spectroscopy (MRS) in the human cortex reported lactate increments of 50–250% [16, 17]. After years of methodological developments in MRS, stimulation of cortical activity has been found to result in lactate concentration increases that do not surpass 30% (e.g [18–23]., reviewed in [24]). Such studies have also found activation-induced increased glutamate that is indicative of fast net glutamate synthesis, reduced glucose due to limited acute brain glucose transport regulation, and reduced aspartate that suggests slow activation of glial pyruvate carboxylation for *de novo* glutamate synthesis (reviewed in [24]). These concentration changes have been partly confirmed in animal models (e.g., [25, 26] reviewed in [27]), and are overall compatible with a coupling between glucose oxidation and glutamate synthesis, which has been determined by ^13^C nuclear magnetic resonance (NMR) spectroscopy in the living brain across various brain activity states [13].

The BOLD effect is a universal observation but the uncoupling between CMR_glc_ and CMR_O2_ is not [15]. An early ^13^C NMR spectroscopy study suggested large stimulation-induced changes in CMR_O2_ (~ 250%) in the anesthetized rat brain [28], suggesting that it is unlikely that lactate concentration increases substantially during brain activation. More recent ^13^C NMR spectroscopy studies using high magnetic fields, have determined increases in CMR_glc_ and CMR_O2_ during somatosensory stimulation in the order of 19–25% and 14–15% in the cortex of rats [29] and tree shrews [26], respectively. These estimations are consistent with expected activity-linked small lactate concentration changes observed in the human brain [24]. An important limitation is that studies in animal models have been mostly conducted under anesthesia, and thus they are not directly comparable to metabolite concentrations determined in awake humans.

In sum, glycolysis is preferentially upregulated during brain activation even though oxygen availability is sufficient (the so-called “aerobic glycolysis”), and the stimulation of glucose oxidation mainly serves to support glutamatergic activity. Fueling of GABA synthesis accounts for about 20% of glucose utilization at rest [30–35], but a coupling between cell-specific energy metabolism fluxes and GABAergic transmission remains to be experimentally determined.

### ^13^C Tracing Experiments for Assessing Metabolic Rates

The most widely used non-invasive method to image neuronal activity is BOLD-fMRI, which relies on measuring the hemodynamic consequences of altered electrical activity. In-between the vascular reactivity and neuronal conduction of electrical impulses, there are changes in energy metabolism that are compartmentalized between neurons and astrocytes, but are not assessable through common measurements of glucose phosphorylation or O_2_ consumption by PET scanning. In turn, NMR spectroscopy coupled to ^13^C tracing constitutes a unique non-invasive, quantitative approach for providing insight into the compartmentalized nature of brain energy metabolism (e.g,. [28, 32, 36–38]).

**Fig. 1:**
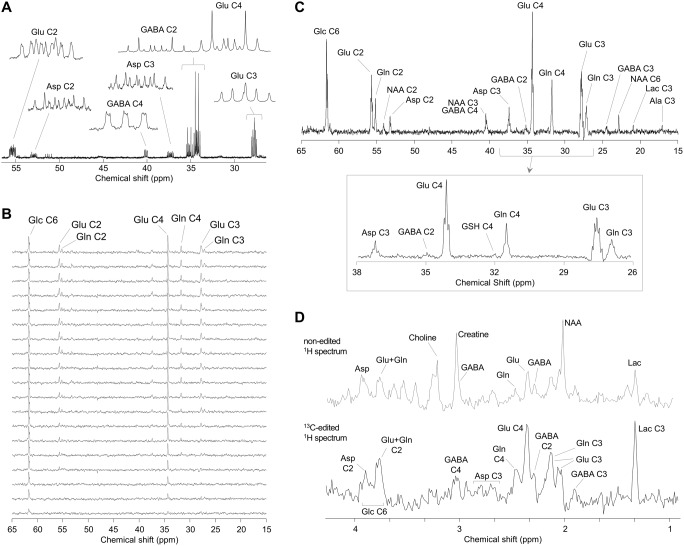
^13^C NMR spectroscopy. ^13^C (**A**) NMR spectrum of an extract from rat hippocampal slices superfused with [2-^13^ C]acetate and [U-^13^C]glucose for 3 h. Spectrum expansions show the multiplets resulting from ^13^C homonuclear coupling. For experimental details see [43]. (**B**) ^13^C spectra acquired from the rat brain during infusion of [1,6-^13^ C]glucose using a semi-adiabatic DEPT sequence (details in [37]). The time course is represented from bottom to top, each spectrum is a 5.3-minute acquisition. (**C**) Spectrum from the same experiment acquired for 1.8 h, starting after 3.5 h of [1,6-^13^ C]glucose infusion. The expansion highlights the presence of multiplets resulting from labeling of adjacent carbons. (**D**) Non-edited and edited spectra from an ^1^H-[^13^C] NMR experiment acquired during 11 min in the mouse hypothalamus (8.7 µL voxel) after 150 min of continuous [1,6-^13^ C]glucose infusion. Experimental details are found elsewhere [44]. Legend: Ala, alanine; Asp, aspartate; Gln, glutamine; Glu, glutamate; Lac, lactate; NAA, *N*-acetylaspartate

One classical approach is referred to as isotopomer analysis, which measures isotopomer (isomers with isotopic atoms) distribution from ^13^C to ^13^C multiplets at a given time point. This approach is widely used for studying tissue extracts and, when combined with ^13^C examination by NMR spectroscopy (Fig. 1A), it is outstanding for determining substrate competition for mitochondria oxidation [39]. Isotopomer analysis has been widely used for studying energy metabolism in brain tissue slices [40, 41] and cells [9, 42].

A second commonly used approach consists of comparing relative ^13^C enrichment from a single ^13^C-enriched substrate into different molecules. Since glucose is the main brain energy substrate, infusion of ^13^C-labeled glucose has been the preferred approach for energy metabolism studies in vivo, coupled with either dynamic ^13^C measurement by either direct ^13^C detection [32, 37] or detection of protons bound to ^13^C atoms (also called ‘indirect ^13^C detection’ or ^1^H-[^13^C] NMR spectroscopy; e.g., [44–46]; Fig. 1B-D).

The strengths of the two approaches have been combined for non-invasive, time-resolved detection of ^13^C-^13^C multiplets in vivo [47, 48]. Despite being technically challenging, this dynamic isotopomer analysis affords a precision increase in the estimation of fluxes associated with glutamatergic activity [48], and has high potential to comprehensively depict metabolic consequences of cerebral activation.

Mathematical modeling is required to determine absolute fluxes of energy metabolism from ^13^C NMR spectroscopy data measured in vivo. The challenges of these modeling approaches have been discussed elsewhere [49]. Current mathematical models of compartmentalized brain energy metabolism that deal with dynamic ^13^C labeling can be used to estimate metabolic fluxes in astrocytes, glutamatergic neurons and GABAergic neurons within a single ^13^C tracing experiment in vivo [30, 32, 34], and efforts have been made to maximize the sensitivity of ^13^C detection in vivo [37, 50]. By increasing sensitivity, as well as amount and precision of experimental data, it has been possible to reduce the number of biochemical assumptions and unknown variables needed for the mathematical modeling process [32, 37, 48].

Absolute fluxes of brain energy metabolism have been determined in vivo with ^13^C NMR spectroscopy using not only glucose as metabolic tracer, but also ^13^C-labeled substrates that are taken by the brain with much smaller avidity, such as lactate [51, 52] or acetate [46, 53, 54].

### Compartmentalized Glucose Oxidation and the Glutamate-Glutamine Cycle

**Fig. 2:**
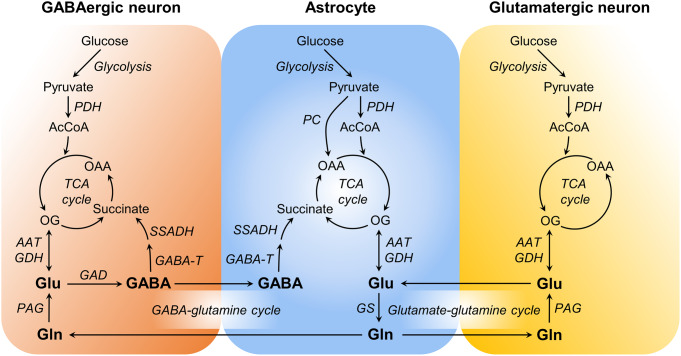
Key metabolic pathways used for modeling glutamatergic and GABAergic energy metabolism from ^13^C MRS data measured in vivo. Cellular energy metabolism is linked to glutamate (Glu) and GABA neurotransmitter cycling between neurons and astrocytes through the glutamate-glutamine cycle and the GABA-glutamine cycle. Glucose is the brain’s primary energetic substrate and is converted to pyruvate through glycolysis. Pyruvate can subsequently enter the mitochondria and be converted to acetyl-CoA (AcCoA) by pyruvate dehydrogenase (PDH) to feed the tricarboxylic acid (TCA) cycle. The TCA cycle intermediate 2-oxoglutarate (OG) is used for glutamate production mainly via aspartate aminotransferase (AAT) and glutamate dehydrogenase (GDH) activity. Astrocytes take up most glutamate and a substantial fraction of GABA from the synapse, leading to depletion of neurotransmitter pools, which are replenished by the provision of glutamine, which is synthesized by glutamine synthetase (GS) in astrocytes, and converted to glutamate by the phosphate-activated glutaminase (PAG). In GABAergic neurons, glutamate is converted to GABA via glutamate decarboxylase (GAD) activity. In astrocytes, glutamate of synaptic origin is either converted into glutamine or oxidized in the TCA cycle as energetic substrate. In turn, GABA must be oxidized in the TCA cycle of astrocytes after the action of GABA transaminase (GABA-T) and succinic semialdehyde dehydrogenase (SSADH) that yields succinate. Neurons likewise have the capability to oxidize both glutamate and GABA. Astrocytes express the anaplerotic enzyme pyruvate carboxylase (PC) that produces oxaloacetate (OAA) from pyruvate, and it contributes to *de novo* synthesis of glutamate and glutamine

Glucose is the brain’s primary energetic substrate [15]. Pyruvate generated from glycolysis is oxidized in the tricarboxylic acid (TCA) cycle of brain cells. The metabolic compartmentation of these pathways has been reviewed elsewhere [38, 49, 55]. Glutamate is produced from the TCA cycle intermediate 2-oxoglutarate, and the neuronal pool of glutamate constitutes the link between energy metabolism and excitatory neurotransmission. Most glutamate released into the synaptic cleft is taken up by astrocytes, where it can be metabolized, or converted into glutamine that is provided back to neurons. In GABAergic neurons, glutamate is converted to GABA. In contrast to glutamate, most GABA is taken up by neurons. After being released into the synaptic cleft, GABA can also be taken up by astrocytes, where it is oxidized in the TCA cycle. Exchange of the neurotransmitters and glutamine between neurons and astrocytes are called the glutamate/GABA-glutamine cycles (Fig. 2). GABAergic neurotransmission is overall less energetically demanding than glutamatergic activity (see [32], and references therein).

**Fig. 3:**
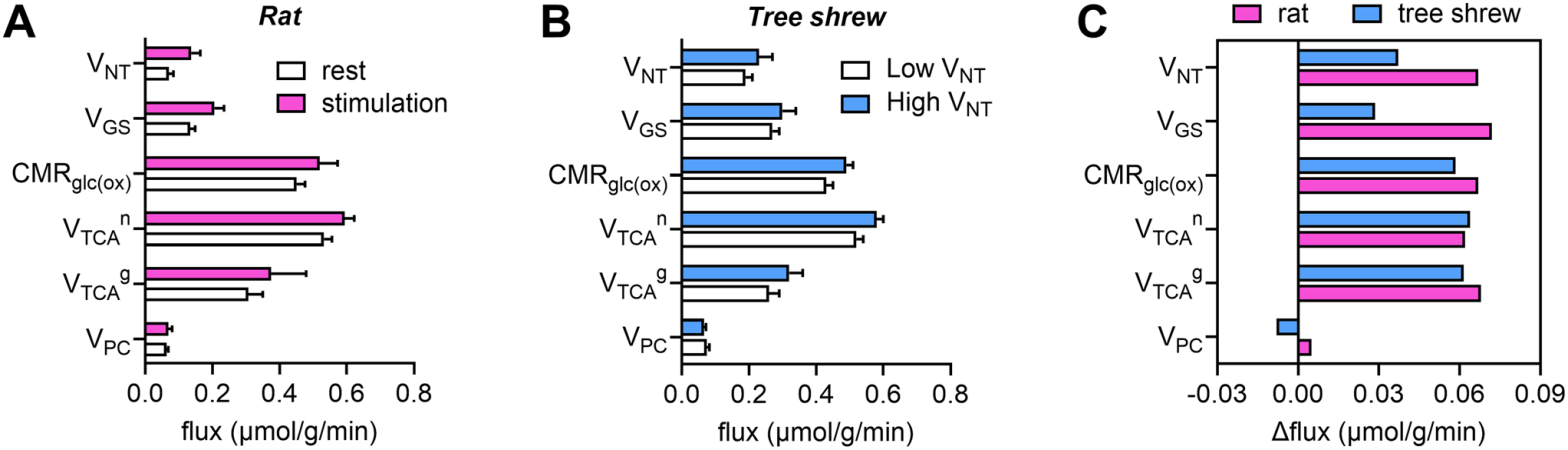
Brain energy metabolism fluxes stimulated by acute cortical activation. Energy metabolism and glutamate-glutamine cycling fluxes estimated at low (rest) and high (stimulation) metabolic activity in the cortex of the rat (**A**) and tree shrew (**B**), previously reported in [29] and [26], respectively. In rats (panel **A**), neuronal stimulation was achieved by electrical stimulation of the four rat paws, and animals were maintained under α-chloralose anesthesia. In tree shrews (panel **B**), a visual stimulation was employed, and anesthesia was light isoflurane. Panel (**C**) depicts the flux variation from low to high cortical activity. The fluxes here depicted are the glutamate-glutamine cycle, taken as apparent glutamatergic neurotransmission (V_NT_), neuronal and astrocytic TCA cycle (V_TCA_^n^ and V_TCA_^g^), pyruvate carboxylase (V_PC_), glutamine synthetase rate (V_GS_ = V_NT_ + V_PC_), the oxidative fraction of CMR_glc_ that is CMR_glc(ox)_ = (V_TCA_^n^ + V_TCA_^g^ + V_PC_)/2. Fluxes are shown in µmol/g/min and error bars represent SD

Glutamatergic transmitter cycling and glucose oxidation are well coupled [13, 38, 56, 57]. ^13^C MRS studies propose that most of the glucose consumption supports glutamatergic transmission. In fact, earlier work already determined a nearly 1:1 relationship between glucose oxidation and glutamatergic neurotransmission, depicted by the glutamate-glutamine cycle rate [58]. These findings have often been interpreted in the context of the proposal that astrocytes are glycolytic cells using two ATPs produced in glycolysis to cover the expense of glutamate uptake and glutamine synthesis, with lactate being produced and made available to supplement extracellular substrate pools (discussed in [15]). Recent ^13^C MRS studies [26, 29] and modeling of available neurochemical data [59] support the notion that astrocytic energy metabolism is oxidative when measured in vivo and is coupled to neuronal function. Accordingly, there is molecular evidence of glutamate transporters GLT-1 and GLAST colocalizing and interacting with Na^+^/K^+^-ATPase and with mitochondria in astrocytic processes [60–64]. Altogether, the studies by Sonnay et al.. showed that variations of neuronal and astrocytic oxidative metabolism are of similar amplitude and coupled to the rate of glutamate-glutamine cycle (Fig. 3A-C), suggesting that increases in astrocytic ATP production extend beyond the immediate needs of glutamate recycling. These studies still support the known stoichiometry between neurotransmission and glucose oxidation.

### Estimating Anaplerosis Via Pyruvate Carboxylation

**Fig. 4:**
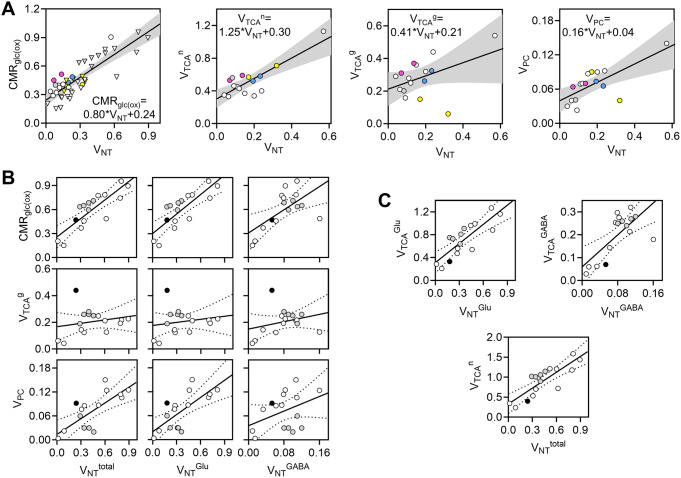
Energy metabolism fluxes coupled to glutamatergic and GABAergic neurotransmission rates. (**A**) Relation between the neurotransmitter cycle rate (V_NT_) and glucose oxidation (CMR_glc(ox)_), neuronal and astroglial TCA cycle (V_TCA_^n^ and V_TCA_^g^), and pyruvate carboxylase flux (V_PC_). Data was collected from several studies using ^13^C NMR spectroscopy and similar compartmentalized mathematical modeling in the brain of rodents, tree shrews (blue symbols) or humans (yellow symbols) reported elsewhere [26, 29, 31, 32, 34, 36, 37, 48, 54, 65, 66–81]. Circles represent studies in which all fluxes in the mathematical model were freely estimated. Triangles represent CMR_glc(ox)_ and V_NT_ pairs estimated from modeling with fixed fluxes (most often V_TCA_^g^ or V_PC_). Pink and blue symbols indicate fluxes from plots in (Fig. 3). (**B**-**C**) Similar correlation analysis was made using metabolic fluxes estimated from experiments in rats that included analysis of ^13^C in GABA using a 3-compartment model of brain metabolism. Filled symbols are from rats under α-chloralose anesthesia, using a model as represented in Fig. 2, and without assumptions on the relation between estimated metabolic fluxes [32]. Open and gray circles are from the brain of rats and mice, respectively [30, 31, 34, 78, 80, 81]. In plots of the 3-compartment model (**B**-**C**), V_TCA_^n^ = V_TCA_^Glu^ + V_TCA_^GABA^ and V_NT_^total^ = V_NT_^Glu^ + V_NT_^GABA^, and most studies assumed astroglial fluxes to be as follows: V_TCA_^g^ was 15% of total TCA cycle flux; V_PC_ was 20% of the glutamine synthesis rate. CMR_glc(ox)_ is the oxidative fraction of CMR_glc_, calculated as follows CMR_glc(ox)_ = (V_TCA_^n^ + V_TCA_^g^ + V_PC_)/2. Fluxes are shown in µmol/g/min. Solid lines represent a linear fit between the 2 fluxes in each plot. The shaded area and dotted lines represent the 95% confidence interval of the linear fit. Data and experimental details are in Supplementary Table S1

Sonnay et al.. provided insight into our understanding of how brain energy metabolism is coupled to glutamate release from neurons, and the rate of the glutamate-glutamine cycle (called V_NT_^glu^), and have also instigated further research questions. In particular, oxidative metabolism in neurons and astrocytes (depicted by V_TCA_) was coupled to V_NT_^glu^ in the cortex upon acute somatosensory stimulation, but astrocyte pyruvate carboxylation (V_PC_) was not (Fig. 3C) [26, 29]. Pyruvate carboxylation activity resides in astrocytes but is lacking in neurons, and is required for de novo neurotransmitter synthesis, namely glutamate. The lack of coupling between V_NT_^glu^ and anaplerotic V_PC_ in acute stimulation contradicts estimations from experiments across multiple activity states (discussed in [38]) (Fig. 4A), a paradox that remains unsolved [57]. Note that there are many published studies included in the plot of CMR_glc(ox)_versus V_NT_ that did not freely estimate astroglial metabolic fluxes (see Supplementary Table S1). Thus, these studies are not suitable for the analysis of V_TCA_^g^ and V_PC_, although they are included in the exercise made in Fig. 4B-C. It is important to note that, when including only studies that did not fix V_TCA_^n^, V_TCA_^g^ and/or V_PC_, the correlations in Fig. 1A are importantly driven by the study in awake rats by Oz et al. [65]. Further studies with minimal modeling constraints are needed. It is also interesting to note that astroglial fluxes from human studies tend to deviate from the pattern observed for animal models (Fig. 4A).

V_PC_ can be detected by distinguishing the amount of ^13^C labeling in carbons C2 and C3 of glutamate and glutamine, unless uniformly labeled glucose is used as tracer [49]. An important issue with estimating V_PC_ from these ^13^C datasets in vivo is its underestimation due to back-flux of labeling from oxaloacetate to fumarate in the TCA cycle that, which leads to ^13^C scrambling between these carbons due to the symmetry of TCA cycle intermediates. When modeling ^13^C traces from brain amino acids, the inclusion of a flux through fumarase (V_fum_), can be detected but is poorly determined, i.e., not significantly different from zero [37, 65]. The inclusion of V_fum_ in the metabolic model results in very small increases in estimated V_PC_. A small pyruvate recycling via malic enzyme activity is known to exist in the brain, but it is undetectable in these experiments in vivo (discussed in [37]). Scrambling of unlabeled HCO_3_ with H^13^CO_3_ eventually released by decarboxylation reactions can also contribute to the uncertainty of V_PC_ estimation during long ^13^C-tracer infusions.

For studying regional rates of V_PC_ in awake rats, McNair et al.. have conducted a study using either [1-^13^ C]glucose or [2-^13^ C]glucose combined with ^1^H-[^13^C] NMR spectroscopy to analyze brain extracts [34].^1^ This interesting experiment did not test acute increases in brain activity, but indicates, as expected, a correlation between V_PC_ and V_NT_ in measurements across different brain areas. Important considerations need to be, however, taken when interpreting the data reported by McNair et al..: the ^13^C fractional enrichment in brain amino acids was below 8% when using [2-^13^ C]glucose as tracer, resulting in poor detection sensitivity; glutamine C2 labeling, which is important for determining V_PC_ from [1-^13^ C]glucose experiments was not analyzed; the modeling approach included several fixed fluxes that might vary across brain areas and experimental conditions, and many parameter constraints that limit the number of fluxes that are freely estimated.

Further studies with minimal modeling constraints are warranted since fixing constraints to metabolic fluxes in astrocytes prevents their free estimation and therefore a real understanding of the metabolism compartmentation. In turn, fitting an excessive number of parameters to the available neurochemical data (i.e., overfitting) results in poorly estimated parameters. One needs to remember that increasing the number of fitted parameters in mathematical models needs to be accompanied by increasing the amount of neurochemical information that is measured and that reflects the respective metabolic fluxes. That means measuring more labeling carbon curves and eventually more metabolites within the pathways in question.

### The GABA-Glutamine Cycling

**Fig. 5:**
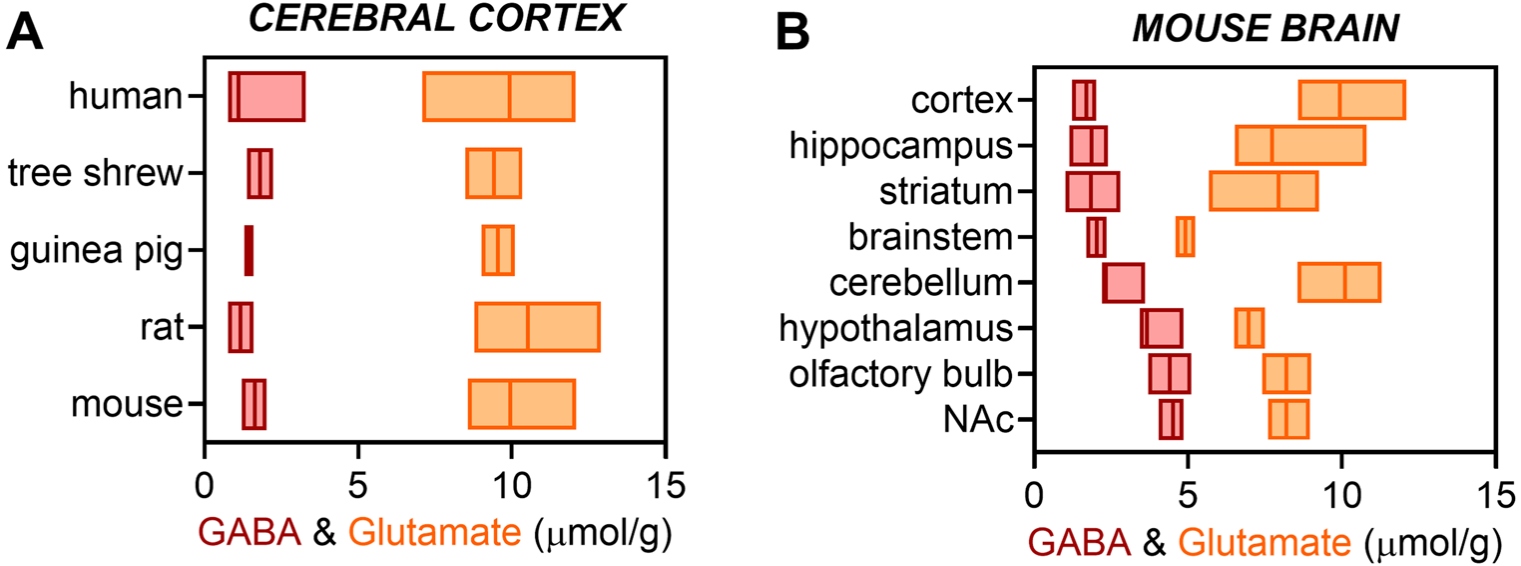
Range of MRS-detected levels of GABA (red) and glutamate (orange) in the cortex of various mammals (**A**) and across regions of the mouse brain (**B**). Plotted concentration ranges and median lines are based on the concentrations reported in ^1^H MRS studies in humans [82–86], tree shrews [26], guinea pigs [87], rats [29, 88–93], and mice [44, 94–96, 97, 98–107]. In these studies, neurotransmitter concentrations were estimated using water concentration as internal reference. Abbreviation: NAc, nucleus accumbens

There is less energy demand for GABA re-uptake as compared to glutamate (see [32], and references therein). Nevertheless, it is of interest to determine the coupling between energy metabolism fluxes and the GABAergic neurotransmission. In contrast to the glutamate-glutamine cycle, it remains challenging to determine the relationship between glucose oxidation and GABAergic transmission because of the relatively small GABA concentrations (Fig. 5), leading to less ^13^C signal in GABA than in glutamate (see [32], and references therein). This precludes high temporal resolution in the measurement of ^13^C signals in vivo. There is hitherto one single study that analyzed ^13^C traces of GABA as measured in vivo using a 3-compartment model as depicted in Fig. 2 [32]. For increasing sensitivity to 13 C detection, NMR spectroscopy was conducted in the whole rat brain at high magnetic field (14.1 T). Following attempts to implement similar analysis in mice failed to estimate fluxes in the astroglial compartment (fixed V_TCA_^g^ and V_PC_) [33, 35]. Other studies have conducted ex vivo analyses of brain extracts after infusion of ^13^C-labeled substrates, and applied a similar modeling approach, although constraining the fitting of the mathematical model with many (perhaps unnecessary) parameter constraints (fixed, not iterated fluxes) based on neurochemical evidence from distinct experimental settings (e.g., [30, 34]). Together, correlations between fluxes estimated from these studies seem to indicate that the GABA-glutamine cycle is correlated with V_PC_ but not V_TCA_^g^ (Fig. 4B). V_PC_ is often fixed to a fraction of glutamine synthesis (see Supplementary Table S1), and then it is definitively correlated with V_NT_. It is also noticeable that parameter constraints lead to underestimation of V_TCA_^g^. Metabolic flux correlations in Fig. 4B are somehow driven by data obtained from models that have not freely estimated either astroglial flux, but rather constrained them to fractions of either neuronal TCA cycle of glutamine synthase. Importantly, in the absence of fitting constraints, V_TCA_^g^ was estimated to be ~ 1/3 of total glucose oxidation, which is much more than what has been assumed in many studies (Fig. 4B). In the neuronal compartment, these studies together suggest that both GABAergic and glutamatergic neurotransmission positively corelate with CMR_glc(ox)_ or the respective neuronal TCA cycles cycle flux (Fig. 4C). Future studies need to improve the metabolic modeling approach by minimizing the number of parameter constraints during model fitting to the ^13^C traces.

Some brain areas have relatively larger amounts of GABA (Fig. 5), namely the rodent nucleus accumbens [94, 95], hypothalamus [44, 45] and olfactory bulb [46, 96]. However, their small size also results in poor direct detection in NMR studies in vivo. In the case of the hypothalamus, sensitivity is further impacted by the distance to the surface of the head, and proximity to the trachea can result in substantial artifacts in the NMR signal (discussed in [108]). Nevertheless, ^13^C NMR spectroscopy has been conducted in the mouse hypothalamus in vivo [44, 45], as well as in the olfactory bulb [46].

### Studying Hypothalamic Metabolism by ^13^C NMR Spectroscopy

Despite being small, the mouse hypothalamus can be easily activated by nutrient signals [108] and is promising for studying how astrocytic metabolism supports GABAergic neurons [45]. At the same time, using a substrate that acts as both metabolic tracer and as hypothalamic stimulus, such as glucose would be, is likely to result in difficult data interpretation, and alternative ^13^C tracing strategies need to be devised. Hypothalamic astrocytes also sense metabolic cues, a function that is likely lost due to inflammation-triggered astrogliosis upon high-fat diet feeding [109]. Diets rich in fat induce alterations of hypothalamic metabolism in mice [45, 97], and impair hypothalamic BOLD responses to glucose after only a week of feeding [108]. The correlation between neuronal and astrocytic metabolic fluxes coupled to glutamatergic and GABAergic neurotransmission has not yet been studied in activated hypothalamus.

By detecting the protons bound to ^13^C carbons using ^1^H-[^13^C] NMR spectroscopy, Lizarbe et al.. measured ^13^C traces of glutamate, glutamine and GABA in the mouse hypothalamus, and estimated a global TCA cycle rate, and rates of GABA and glutamine synthesis, using a single compartment model [44, 45]. A limitation of using indirect ^13^C detection for increasing sensitivity is the inability to simultaneously determine labeling in C2 and C3 of both glutamate and glutamine, which is a critical feature for robustly distinguishing astrocytes from neurons as two independent metabolic compartments. Therefore, measuring fluxes in three metabolic compartments and obtaining fluxes of energy metabolism in astroglia, glutamatergic neurons and GABAergic neurons has not been yet possible.

### Direct *Versus* Indirect Detection of ^13^C

The ^13^C enrichment of brain metabolites measured in vivo is largely restricted to the most abundant amino acids and neurotransmitters (glutamate, glutamine, aspartate and GABA) due to low signal-to-noise ratio, which is partly due to the less favorable magnetic properties of ^13^C compared to ^1^H, namely lower gyromagnetic ratio and typically long T_1_ relaxation. In turn, there are experimental restrictions in terms of the spatial and temporal resolutions achievable in the living brain (e.g. Sonnay et al.. have used a small volume of interest of ~ 100 mm^3^ [26]). For increasing sensitivity, it is indeed advantageous to detect ^1^H-bound ^13^C carbons in proton spectra by ^1^H-[^13^C] NMR spectroscopy. The increase in sensitivity for ^13^C labeling detection in the brain allows the conduction of ^13^C tracing experiments in small brain volumes such as the mouse hypothalamus [44, 45] or olfactory bulb [46]. However, this technique has the disadvantage of the intrinsically lower spectral resolution of ^1^H compared to ^13^C NMR spectroscopy and, therefore, limited number of carbons that can be effectively detected. For example, even at high magnetic field (14.1 T), ^1^H-[^13^C] NMR spectroscopy did not allow to separate labeling of glutamate C2 from that of glutamine C2 [53], which are two key carbons to determine the rate of pyruvate carboxylation, and therefore to distinguish the compartmentation between astrocytes and neurons.

### Hyperpolarized ^13^C MRS for Metabolism Studies

Dynamic nuclear polarization (DNP) and hyperpolarized ^13^C NMR spectroscopy has emerged as a technique that may overcome some of these spatial and temporal limitations of ^13^C tracing experiments in vivo. It does overcome the limitations of ^13^C NMR spectroscopy by providing a transient increase in signal-to-noise ratio of at least 10,000-fold [110], allowing the uptake and metabolic conversion of physiologically relevant substrates to be measured in the living brain at temporal resolutions below 1 s [111], although 2–5 s measurements are more common (e.g [112, 113]).

Brain energy metabolism measured using hyperpolarized NMR spectroscopy remains hitherto largely limited to substrate utilization and production of pyruvate, lactate, alanine and/or bicarbonate production after injection of hyperpolarized [1-^13^ C]pyruvate or [1-^13^ C]lactate (e.g., [112, 114, 115]). This production of [^13^C]bicarbonate can be used to estimate pyruvate dehydrogenase activity, but it does not necessarily represent the actual TCA cycle rate. Instead, the use of hyperpolarized [2-^13^ C]pyruvate can be used for the in vivo investigation of the TCA cycle in the brain by producing [1-^13^ C]acetylcarnitine, [1-^13^ C]citrate and [5-^13^ C]glutamate ([1-^13^ C]acetoacetate signal was also detected [116]). A study using [2,3,4,6,6-^2^H_5_, 3,4-^13^C_2_]glucose as substrate has detected 3-phosphoglycerate, [1-^13^ C]pyruvate, and [1-^13^ C]lactate, which allows determining the cerebral metabolic rate of glucose that is often assessable by ^18^F-fluorodeoxyglucose (^18^FDG) PET [111]. However, it does not allow to determine oxidative metabolism rate, that is, the TCA cycle rate. Recently, using hyperpolarized [^2^H_7_, U-^13^C]glucose and a combination of ^2^H and ^13^C NMR spectroscopy, Flatt et al.. measured the formation of lactate under isoflurane anesthesia (widely used anesthesia protocol in rodent studies), which was absent when animals were sedated with medetomidine and with isoflurane supplemented below 0.5% [117]. It is known that isoflurane increases the pool of brain lactate [118], which certainly contributed to the visibility of lactate in the measured spectra. The interesting finding of Flatt’s study was that, using medetomidine and thus reducing lactate formation, it was possible to observe a signal that corresponds to glutamate (and/or glutamine) using ^2^H detection. Although ^2^H spectroscopy did not provide spectral resolution to determine the rate of glutamate labeling, technical refinements in future studies can allow to determine rates of mitochondrial metabolism from hyperpolarized glucose.

By using hyperpolarized [1-^13^ C]acetate or [1,2-^13^C_2_]acetate and ^13^C NMR spectroscopy, Mishkovsky et al.. detected the formation of [5-^13^ C]2-oxoglutarate and [4,5-^13^C_2_]2-oxoglutarate, respectively [119]. Since acetate is mainly metabolized in astrocytes (e.g., discussed in [57]), this approach can be used to determine the TCA cycle rate in this cell type. In the study by Mishkovsky et al.., given that astrocytic glutamate is at least one order of magnitude more abundant than 2-oxoglutarate [49], the lack of labeling appearance in glutamate suggests that transamination of 2-oxoglutarate and glutamate formation in astrocytes is much slower than the TCA cycle rate.

In sum, while hyperpolarized NMR spectroscopy allows for increasing ^13^C sensitivity, it has been rarely used to determine oxidative metabolism rates and, at this moment, it does not allow to investigate cellular compartmentation of energy metabolism. However, astrocytic energy metabolism can be specifically probed by employing ^13^C-labeled acetate.

### Brain Energy Metabolism Probed with ^2^H MRS

Deuterium (^2^H) is magnetically active hydrogen isotope, and it has been used to probe brain energy metabolism in two modalities. First, direct detection of ^2^H spectra can reveal metabolism of deuterated glucose or other substrates (acetate and β-hydroxybutyrate have also been used) into brain amino acids [117, 120–122]. This approach is proposed to provide an alternative to ^18^FDG PET imaging that goes beyond glucose uptake [121]. Upon administration of [6,6′-^2^H_2_]glucose, it has been possible to determine the appearance of labeling in brain glucose, glutamate plus glutamine, and lactate in vivo [117, 120, 121]. However, the poor spectral resolution of ^2^H spectra is far from allowing investigation of metabolic compartmentation in a single experiment. Second, the labeling from deuterated substrates into amino acids can be followed by the signal difference in ^1^H spectra [123, 124]. This approach provides the sensitivity of ^1^H MRS for detection of glutamate, glutamine, GABA and lactate, but has the drawback of peak overlapping already mentioned above for indirect ^13^C detection with ^1^H-[^13^C] NMR spectroscopy, and deuterated acetate tracers, such as [^2^H_3_]acetate, could be used to specifically probe astrocytic metabolism [121, 123].

### Levels of Glutamate and GABA Measured by ^1^H MRS

Although metabolic compartmentation is not addressed by ^1^H NMR spectroscopy measurement of metabolite levels, such studies can inform on metabolite associations with brain function. Over the last decades, the availability of high-field magnets and several technical developments in data acquisition and analysis allowed increasing resolution and sensitivity of both ^1^H NMR spectroscopy and functional imaging. Then several studies provided evidence for a relation between brain function and metabolite levels, namely the neurotransmitters glutamate and GABA. The metabolite profile changes during activation and deactivation in of the human cortex have been reviewed recently [24]. Most ^1^H NMR spectroscopy studies showed a small but robust activation-induced increase in the concentration of glutamate, and a decrease in the concentration of GABA that seems to depend on brain region analyzed and stimulus employed [24, 125]. Concentrations of glutamate and GABA in the human cortex are also reported to decrease during deactivation, that is, during negative BOLD responses in MRI [21].^2^ The activity-associated glutamate increase has been confirmed during visual or somatosensory stimulation in animal models (e.g [25, 26, 129]). and consistently reproduced across several studies in rodents (reviewed in [27]). The activity-associated variations of GABA levels remain to be robustly shown in animal models. The most important limitation for conducting such functional NMR spectroscopy studies is the utilization of anesthesia, which alters not only neuronal activity but also concentrations of glutamate and GABA at rest [118]. The development of multimodal anesthesia protocols that combine two or more complementary drugs can eventually result in specific brain states during MRS studies [130]. Recently, Takado et al.. reported that somatosensory stimulation in awake mice leads to an increase in the cortical concentrations of both glutamate and GABA [131]. However, the stress involved in such an experimental approach needs to be considered [130]. In addition, these GABA changes reported by Takado et al.. show considerable variability that might not be caused by biological differences between animals. Accuracy in GABA quantification could be improved by employing ^1^H MRS with spectral editing [132, 133].

Besides the direct modulation of glutamate and GABA by brain activity, it is also reported that baseline levels of glutamate are positively associated with the strength of neuronal connectivity in the brain, while the opposite (negative association) is generally found for GABA levels [134–136]. In addition, resting GABA levels are negatively correlated to stimulus-induced brain activation [136].

These associations between functional outcomes and metabolite levels or metabolite concentration changes are interesting, but do not provide information on the flux through metabolic pathways that are activated or repressed in neurons and astrocytes during brain activation/deactivation. This highlights the value of ^13^C NMR spectroscopy studies.

### A Note on the Significance of Studying GABAergic Metabolism

Non-invasive ^13^C MRS is a unique methodology that allows translational studies in models and humans but is, however, not accessible to many labs across the world. Nevertheless, this methodology allows the advancing of our knowledge on neuron-glia cooperation, namely on the relations between astrocytic metabolism and glutamatergic and GABAergic activity in the living brain. While most studies have, mainly due to technical limitations, exclusively focused on glutamate-glutamine cycling, studying GABAergic metabolism would also contribute to decipher the neuroenergetic and neurochemical basis of brain activation in the context of excitatory-inhibitory balance. Importantly, neurodegnerative or psychiatric disorders have a non-negligible metabolic component, and rescuing energy metabolism is proposed to improve brain health [137–139]. Typifying the importance of MRS-detected glutamate and GABA for cognition, resting glutamate but not GABA concentration in the rat hippocampus was found associated with performance in a hippocampal-dependent memory task [140]. In humans, recall of a visual cue from a paired associate also elicited a transient increase in the ratio between glutamate and GABA in visual cortex [141].

Developing methodology to study the hypothalamus with high sensitivity is of unique importance since (i) the hypothalamus is rich in GABA, thus providing high sensitivity to study GABAergic metabolism; (ii) the negative BOLD response in the hypothalamus [108, 142] is likely associated to GABAergic activity^3^; (iii) it can enhance our understanding of hypothalamic activity in obesity and metabolic disease, which involve dysfunction of metabolic sensors in hypothalamic nuclei (e.g., [44, 97, 108, 109]).

## Conclusion

Even though the core biochemistry of the TCA cycle was elucidated a long time ago, our understanding of the regulation and function of the TCA cycle in a cell-specific manner is not fully understood and further research is warranted [144]. Neurons and astrocytes show divergent TCA cycle participation in the synthesis and oxidation of glutamate and GABA [55], but how this divergence is achieved and the extent of how such cell-specific regulation contributes to support neurotransmitter release and cycling during stimulation of neuronal activity remains largely unknown [57].

Being metabolic supporters of neuronal activity, astrocytes also sense signals from traditional neuromodulators, and adjust their metabolism accordingly. For example, the control of glucose utilization, glycogen metabolism and TCA cycle in astrocytes is reported to be modulated by endocannabinoids that are produced postsynaptically [40, 145, 146], and adenosine produced extracellularly from the ATP co-released with neurotransmitters [41, 147, 148]. These and other molecular controllers of the compartmentalization of energy metabolism deserve further research, and ^13^C tracing coupled to NMR spectroscopy in vivo is particularly suited for such studies. However, NMR spectroscopy alone will not provide fine details on metabolic dynamics at cellular level, and it should be combined with emerging tools such as cell-specific optogenetic control, and the use of genetically-encoded fluorescent indicators that are capable of monitoring brain metabolites in a compartmentalized fashion (reviewed elsewhere [149]). Indeed, optogenetic fMRI has been employed to disentangle the specific role of various cells within neuronal networks [150, 151], and optogenetic MRS is being implemented successfully [152].

## Electronic Supplementary Material

Below is the link to the electronic supplementary material.

Supplementary Material 1

## Data Availability

No datasets were generated or analysed during the current study.

## Footnotes

1 [1-^13^ C]glucose carbons that enter the TCA cycle via pyruvate dehydrogenase give rise to C4-labeled 2-oxoglutarate (and then glutamate and glutamine C4, and GABA C2). In turn, In turn, [2-^13^ C]glucose gives rise to 2-oxoglutarate C5 (and then glutamate and glutamine C5, and GABA C1). Following metabolism through subsequent turns of the TCA cycle, labeling from [1-^13^ C]glucose but not from [2-^13^ C]glucose will appear in glutamate and glutamine C2 and C3, as well as GABA C3 and C4. These carbons will only become labeled from [2-^13^ C]glucose when pyruvate is metabolised via V_PC_ (see details in [34]).

2 The negative BOLD response is a prevalent feature of brain activity during cognitive tasks or sensory stimuli. Its association with neuronal inhibition is generally accepted. It is thought to reflect suppression or deactivation of cortical areas unrequired for task performance, but much remains to be understood regarding its response properties and generative pathways [126]. In the human occipital cortex, the negative BOLD is temporally and spatially linked to blood flow decreases. By modelling cortical BOLD and blood flow responses based on cortical energy consumption, the Shmuel et al.. suggested that this negative BOLD response is primarily caused by neuronal inhibition, and reductions in oxygen consumption [127]. Still, GABA concentration is reduced during a negative BOLD response [21]. There are hitherto no reports of increased GABA levels. Despite reduced GABA levels, GABAergic stimulation has been linked to the negative BOLD response. In turn, Walter et al.. reported that BOLD responses in the cingulate cortex were inversely correlated with blood levels of the positive allosteric modulator of GABA diazepam, but not overlapping with task-related negative BOLD [128].

3 While negative BOLD responses have been associated with activity of inhibitory neurons [127], negative BOLD changes have been reported upon stimulation of not only the hypothalamus [108, 142] but also the hippocampus (Schridde et al., 2008) [143].

## References

[1] Andersen JV, Schousboe A, Milestone, Review (2023) Metabolic dynamics of glutamate and GABA mediated neurotransmission - the essential roles of astrocytes. J Neurochem 166(2):109–137. 10.1111/jnc.15811Epub 2023 Mar 29. PMID: 36919769 36919769 10.1111/jnc.15811

[2] McKenna MC, Stridh MH, McNair LF, Sonnewald U, Waagepetersen HS, Schousboe A (2016) Glutamate oxidation in astrocytes: roles of glutamate dehydrogenase and aminotransferases. J Neurosci Res 94(12):1561–1571. 10.1002/jnr.23908Epub 2016 Sep 15. PMID: 27629247 27629247 10.1002/jnr.23908

[3] Son H, Kim S, Jung DH, Baek JH, Lee DH, Roh GS, Kang SS, Cho GJ, Choi WS, Lee DK, Kim HJ (2019) Insufficient glutamine synthetase activity during synaptogenesis causes spatial memory impairment in adult mice. Sci Rep 9(1):252. 10.1038/s41598-018-36619-2 30670758 10.1038/s41598-018-36619-2 PMC6342969

[4] Zehnder T, Petrelli F, Romanos J, De Oliveira Figueiredo EC, Lewis TL Jr, Déglon N, Polleux F, Santello M, Bezzi P (2021) Mitochondrial biogenesis in developing astrocytes regulates astrocyte maturation and synapse formation. Cell Rep 35(2):108952. 10.1016/j.celrep.2021.108952 33852851 10.1016/j.celrep.2021.108952

[5] Andersen JV, Christensen SK, Westi EW, Diaz-delCastillo M, Tanila H, Schousboe A, Aldana BI, Waagepetersen HS (2021) Deficient astrocyte metabolism impairs glutamine synthesis and neurotransmitter homeostasis in a mouse model of Alzheimer’s disease. Neurobiol Dis 148:105198. 10.1016/j.nbd.2020.105198 33242587 10.1016/j.nbd.2020.105198

[6] Saab AS, Tzvetavona ID, Trevisiol A, Baltan S, Dibaj P, Kusch K, Möbius W, Goetze B, Jahn HM, Huang W, Steffens H, Schomburg ED, Pérez-Samartín A, Pérez-Cerdá F, Bakhtiari D, Matute C, Löwel S, Griesinger C, Hirrlinger J, Kirchhoff F, Nave KA (2016) Oligodendroglial NMDA Receptors Regulate Glucose Import and Axonal Energy Metabolism. Neuron 91(1):119–132. 10.1016/j.neuron.2016.05.016 27292539 10.1016/j.neuron.2016.05.016 PMC9084537

[7] Looser ZJ, Faik Z, Ravotto L, Zanker HS, Jung RB, Werner HB, Ruhwedel T, Möbius W, Bergles DE, Barros LF, Nave KA, Weber B, Saab AS (2024) Oligodendrocyte-axon metabolic coupling is mediated by extracellular K + and maintains axonal health. Nat Neurosci 27(3):433–448. 10.1038/s41593-023-01558-3 38267524 10.1038/s41593-023-01558-3 PMC10917689

[8] Shen Y, Kapfhamer D, Minnella AM, Kim JE, Won SJ, Chen Y, Huang Y, Low LH, Massa SM, Swanson RA (2017) Bioenergetic state regulates innate inflammatory responses through the transcriptional co-repressor CtBP. Nat Commun 8(1):624. 10.1038/s41467-017-00707-0 28935892 10.1038/s41467-017-00707-0 PMC5608947

[9] De Paula GC, Aldana BI, Battistella R, Fernández-Calle R, Bjure A, Lundgaard I, Deierborg T, Duarte JMN (2024) Extracellular vesicles released from microglia after palmitate exposure impact brain function. J Neuroinflammation 21(1):173. 10.1186/s12974-024-03168-7 39014461 10.1186/s12974-024-03168-7 PMC11253458

[10] Mergenthaler P, Lindauer U, Dienel GA, Meisel A (2013) Sugar for the brain: the role of glucose in physiological and pathological brain function. Trends Neurosci 36(10):587–597. 10.1016/j.tins.2013.07.001 23968694 10.1016/j.tins.2013.07.001 PMC3900881

[11] Fox PT, Raichle ME (1986) Focal physiological uncoupling of cerebral blood flow and oxidative metabolism during somatosensory stimulation in human subjects. Proc Natl Acad Sci U S A 83(4):1140–1144. 10.1073/pnas.83.4.1140 3485282 10.1073/pnas.83.4.1140 PMC323027

[12] Fox PT, Raichle ME, Mintun MA, Dence C (1988) Nonoxidative glucose consumption during focal physiologic neural activity. Science 241(4864):462–464. 10.1126/science.3260686 3260686 10.1126/science.3260686

[13] Hyder F, Rothman DL (2012) Quantitative fMRI and oxidative neuroenergetics. NeuroImage 62(2):985–994. 10.1016/j.neuroimage.2012.04.027 22542993 10.1016/j.neuroimage.2012.04.027 PMC3389300

[14] DiNuzzo M, Dienel GA, Behar KL, Petroff OA, Benveniste H, Hyder F, Giove F, Michaeli S, Mangia S, Herculano-Houzel S, Rothman DL (2024) Neurovascular coupling is optimized to compensate for the increase in proton production from nonoxidative glycolysis and glycogenolysis during brain activation and maintain homeostasis of pH, pCO2, and pO2. J Neurochem 168(5):632–662. 10.1111/jnc.15839 37150946 10.1111/jnc.15839 PMC10628336

[15] Dienel GA (2019) Brain glucose metabolism: integration of energetics with function. Physiol Rev 99(1):949–1045. 10.1152/physrev.00062.2017 30565508 10.1152/physrev.00062.2017

[16] Prichard J, Rothman D, Novotny E, Petroff O, Kuwabara T, Avison M, Howseman A, Hanstock C, Shulman R (1991) Lactate rise detected by 1H NMR in human visual cortex during physiologic stimulation. Proc Natl Acad Sci U S A 88(13):5829–5831. 10.1073/pnas.88.13.5829 2062861 10.1073/pnas.88.13.5829 PMC51971

[17] Sappey-Marinier D, Calabrese G, Fein G, Hugg JW, Biggins C, Weiner MW (1992) Effect of photic stimulation on human visual cortex lactate and phosphates using 1H and 31P magnetic resonance spectroscopy. J Cereb Blood Flow Metab 12(4):584–592. 10.1038/jcbfm.1992.82 1618937 10.1038/jcbfm.1992.82

[18] Mangia S, Tkác I, Gruetter R, Van de Moortele PF, Maraviglia B, Uğurbil K (2007) Sustained neuronal activation raises oxidative metabolism to a new steady-state level: evidence from 1H NMR spectroscopy in the human visual cortex. J Cereb Blood Flow Metab 27(5):1055–1063. 10.1038/sj.jcbfm.9600401 17033694 10.1038/sj.jcbfm.9600401

[19] Schaller B, Mekle R, Xin L, Kunz N, Gruetter R (2013) Net increase of lactate and glutamate concentration in activated human visual cortex detected with magnetic resonance spectroscopy at 7 tesla. J Neurosci Res 91(8):1076–1083. 10.1002/jnr.23194 23378234 10.1002/jnr.23194

[20] Schaller B, Xin L, O’Brien K, Magill AW, Gruetter R (2014) Are glutamate and lactate increases ubiquitous to physiological activation? A (1)H functional MR spectroscopy study during motor activation in human brain at 7Tesla. NeuroImage 93(Pt 1):138–145. 10.1016/j.neuroimage.2014.02.016 24555953 10.1016/j.neuroimage.2014.02.016

[21] Boillat Y, Xin L, van der Zwaag W, Gruetter R (2020) Metabolite concentration changes associated with positive and negative BOLD responses in the human visual cortex: a functional MRS study at 7 Tesla. J Cereb Blood Flow Metab 40(3):488–500. 10.1177/0271678X19831022 30755134 10.1177/0271678X19831022 PMC7026843

[22] Koush Y, de Graaf RA, Kupers R, Dricot L, Ptito M, Behar KL, Rothman DL, Hyder F (2021) Metabolic underpinnings of activated and deactivated cortical areas in human brain. J Cereb Blood Flow Metab 41(5):986–1000. 10.1177/0271678X21989186 33472521 10.1177/0271678X21989186 PMC8054719

[23] DiNuzzo M, Mangia S, Moraschi M, Mascali D, Hagberg GE, Giove F (2022) Perception is associated with the brain’s metabolic response to sensory stimulation. Elife 11:e71016. 10.7554/eLife.71016 35225790 10.7554/eLife.71016 PMC9038191

[24] Koush Y, Rothman DL, Behar KL, de Graaf RA, Hyder F (2022) Human brain functional MRS reveals interplay of metabolites implicated in neurotransmission and neuroenergetics. J Cereb Blood Flow Metab 42(6):911–934. 10.1177/0271678X221076570 35078383 10.1177/0271678X221076570 PMC9125492

[25] Sonnay S, Duarte JMN, Just N (2017) Lactate and glutamate dynamics during prolonged stimulation of the rat barrel cortex suggest adaptation of cerebral glucose and oxygen metabolism. Neuroscience 346:337–348. 10.1016/j.neuroscience.2017.01.034 28153690 10.1016/j.neuroscience.2017.01.034

[26] Sonnay S, Poirot J, Just N, Clerc AC, Gruetter R, Rainer G, Duarte JMN (2018) Astrocytic and neuronal oxidative metabolism are coupled to the rate of glutamate-glutamine cycle in the tree shrew visual cortex. Glia 66(3):477–491. 10.1002/glia.23259 29120073 10.1002/glia.23259

[27] Just N (2021) Proton functional magnetic resonance spectroscopy in rodents. NMR Biomed 34(5):e4254. 10.1002/nbm.4254 31967711 10.1002/nbm.4254

[28] Hyder F, Chase JR, Behar KL, Mason GF, Siddeek M, Rothman DL, Shulman RG (1996) Increased tricarboxylic acid cycle flux in rat brain during forepaw stimulation detected with 1H[13 C]NMR. Proc Natl Acad Sci U S A 93(15):7612–7617. 10.1073/pnas.93.15.7612 8755523 10.1073/pnas.93.15.7612 PMC38794

[29] Sonnay S, Duarte JM, Just N, Gruetter R (2016) Compartmentalised energy metabolism supporting glutamatergic neurotransmission in response to increased activity in the rat cerebral cortex: a 13 C MRS study in vivo at 14.1 T. J Cereb Blood Flow Metab 36(5):928–940. 10.1177/0271678X16629482 26823472 10.1177/0271678X16629482 PMC4853840

[30] Patel AB, de Graaf RA, Mason GF, Rothman DL, Shulman RG, Behar KL (2005) The contribution of GABA to glutamate/glutamine cycling and energy metabolism in the rat cortex in vivo. Proc Natl Acad Sci U S A 102(15):5588–5593. 10.1073/pnas.0501703102 15809416 10.1073/pnas.0501703102 PMC556230

[31] Chowdhury GM, Patel AB, Mason GF, Rothman DL, Behar KL (2007) Glutamatergic and GABAergic neurotransmitter cycling and energy metabolism in rat cerebral cortex during postnatal development. J Cereb Blood Flow Metab 27(12):1895–1907. 10.1038/sj.jcbfm.9600490 17440492 10.1038/sj.jcbfm.9600490

[32] Duarte JM, Gruetter R (2013) Glutamatergic and GABAergic energy metabolism measured in the rat brain by (13) C NMR spectroscopy at 14.1 T. J Neurochem 126(5):579–590. 10.1111/jnc.12333 23745684 10.1111/jnc.12333

[33] Cherix A, Donati G, Lizarbe B, Lanz B, Poitry-Yamate C, Lei H, Gruetter R (2021) Excitatory/inhibitory neuronal metabolic balance in mouse hippocampus upon infusion of [U-13C6]glucose. J Cereb Blood Flow Metab 41(2):282–297. 10.1177/0271678X20910535 32151224 10.1177/0271678X20910535 PMC8370000

[34] McNair LM, Mason GF, Chowdhury GM, Jiang L, Ma X, Rothman DL, Waagepetersen HS, Behar KL (2022) Rates of pyruvate carboxylase, glutamate and GABA neurotransmitter cycling, and glucose oxidation in multiple brain regions of the awake rat using a combination of [2-^13^C]/[1-^13^C]glucose infusion and ^1^H-[^13^C]NMR *ex vivo*. J Cereb Blood Flow Metab 42(8):1507–1523. 10.1177/0271678X221074211 35048735 10.1177/0271678X221074211 PMC9274856

[35] Cherix A, Poitry-Yamate C, Lanz B, Zanoletti O, Grosse J, Sandi C, Gruetter R, Cardinaux JR (2022) Deletion of Crtc1 leads to hippocampal neuroenergetic impairments associated with depressive-like behavior. Mol Psychiatry 27(11):4485–4501. 10.1038/s41380-022-01791-5 36224260 10.1038/s41380-022-01791-5 PMC9734042

[36] Gruetter R, Seaquist ER, Ugurbil K (2001) A mathematical model of compartmentalized neurotransmitter metabolism in the human brain. Am J Physiol Endocrinol Metab 281(1):E100–E112. 10.1152/ajpendo.2001.281.1.E100 11404227 10.1152/ajpendo.2001.281.1.E100

[37] Duarte JM, Lanz B, Gruetter R (2011) Compartmentalized cerebral metabolism of [1,6-(13)C]glucose determined by in vivo 13 C NMR spectroscopy at 14.1 T. Front Neuroenergetics 3:3. 10.3389/fnene.2011.00003 21713114 10.3389/fnene.2011.00003 PMC3112327

[38] Sonnay S, Gruetter R, Duarte JMN (2017) How Energy Metabolism supports cerebral function: insights from 13 C magnetic resonance studies in vivo. Front Neurosci 11:288. 10.3389/fnins.2017.00288 28603480 10.3389/fnins.2017.00288 PMC5445183

[39] Jeffrey FM, Rajagopal A, Malloy CR, Sherry AD (1991) 13 C-NMR: a simple yet comprehensive method for analysis of intermediary metabolism. Trends Biochem Sci 16(1):5–10. 10.1016/0968-0004(91)90004-f 2053137 10.1016/0968-0004(91)90004-f

[40] Duarte JM, Ferreira SG, Carvalho RA, Cunha RA, Köfalvi A (2012) CB₁ receptor activation inhibits neuronal and astrocytic intermediary metabolism in the rat hippocampus. Neurochem Int 60(1):1–8. 10.1016/j.neuint.2011.10.019 22085448 10.1016/j.neuint.2011.10.019

[41] Duarte JM, Cunha RA, Carvalho RA (2016) Adenosine A1 receptors control the metabolic recovery after hypoxia in rat hippocampal slices. J Neurochem 136(5):947–957. 10.1111/jnc.13512 26709861 10.1111/jnc.13512

[42] Amaral AI, Teixeira AP, Håkonsen BI, Sonnewald U, Alves PM (2011) A comprehensive metabolic profile of cultured astrocytes using isotopic transient metabolic flux analysis and 13 C-labeled glucose. Front Neuroenergetics 3:5. 10.3389/fnene.2011.00005 21941478 10.3389/fnene.2011.00005 PMC3171112

[43] Duarte JM, Cunha RA, Carvalho RA (2007) Different metabolism of glutamatergic and GABAergic compartments in superfused hippocampal slices characterized by nuclear magnetic resonance spectroscopy. Neuroscience 144(4):1305–1313. 10.1016/j.neuroscience.2006.11.027 17197104 10.1016/j.neuroscience.2006.11.027

[44] Lizarbe B, Cherix A, Duarte JMN, Cardinaux JR, Gruetter R (2019) High-fat diet consumption alters energy metabolism in the mouse hypothalamus. Int J Obes (Lond) 43(6):1295–1304. 10.1038/s41366-018-0224-9 30301962 10.1038/s41366-018-0224-9

[45] Lizarbe B, Lei H, Duarte JMN, Lanz B, Cherix A, Gruetter R (2018) Feasibility of in vivo measurement of glucose metabolism in the mouse hypothalamus by 1H-[13 C] MRS at 14.1T. Magn Reson Med 80(3):874–884. 10.1002/mrm.27129 29427382 10.1002/mrm.27129

[46] Chowdhury GMI, Behar KL, Mason GF, Rothman DL, de Graaf RA (2024) Measurement of neuro-energetics and neurotransmission in the rat olfactory bulb using 1H and 1H-[13 C] NMR spectroscopy. NMR Biomed 37(6):e4957. 10.1002/nbm.4957 37088548 10.1002/nbm.4957 PMC10590826

[47] Shestov AA, Valette J, Deelchand DK, Uğurbil K, Henry PG (2012) Metabolic modeling of dynamic brain ¹³C NMR multiplet data: concepts and simulations with a two-compartment neuronal-glial model. Neurochem Res 37(11):2388–2401. 10.1007/s11064-012-0782-5 22528840 10.1007/s11064-012-0782-5 PMC4806787

[48] Dehghani MM, Lanz B, Duarte JMN, Kunz N, Gruetter R (2016) Refined Analysis of Brain Energy Metabolism using in vivo Dynamic Enrichment of 13 C multiplets. ASN Neuro 8(2):1759091416632342. 10.1177/1759091416632342 26969691 10.1177/1759091416632342 PMC4790427

[49] Lanz B, Gruetter R, Duarte JM (2013) Metabolic flux and Compartmentation Analysis in the Brain in vivo. Front Endocrinol 4:156. 10.3389/fendo.2013.00156 10.3389/fendo.2013.00156 PMC3809570 24194729

[50] Henry PG, Tkác I, Gruetter R (2003) 1H-localized broadband 13 C NMR spectroscopy of the rat brain in vivo at 9.4 T. Magn Reson Med 50(4):684–692. 10.1002/mrm.10601 14523952 10.1002/mrm.10601

[51] Boumezbeur F, Petersen KF, Cline GW, Mason GF, Behar KL, Shulman GI, Rothman DL (2010) The contribution of blood lactate to brain energy metabolism in humans measured by dynamic 13 C nuclear magnetic resonance spectroscopy. J Neurosci 30(42):13983–13991. 10.1523/JNEUROSCI.2040-10.2010 20962220 10.1523/JNEUROSCI.2040-10.2010 PMC2996729

[52] Duarte JM, Girault FM, Gruetter R (2015) Brain energy metabolism measured by (13)C magnetic resonance spectroscopy in vivo upon infusion of [3-(13)C]lactate. J Neurosci Res 93(7):1009–1018. 10.1002/jnr.23531 25522255 10.1002/jnr.23531

[53] Xin L, Mlynárik V, Lanz B, Frenkel H, Gruetter R (2010) 1H-[13 C] NMR spectroscopy of the rat brain during infusion of [2-13 C] acetate at 14.1 T. Magn Reson Med 64(2):334–340. 10.1002/mrm.22359 20535808 10.1002/mrm.22359

[54] Lanz B, Xin L, Millet P, Gruetter R (2014) In vivo quantification of neuro-glial metabolism and glial glutamate concentration using 1H-[13 C] MRS at 14.1T. J Neurochem 128(1):125–139. 10.1111/jnc.12479 24117599 10.1111/jnc.12479

[55] Andersen JV, Markussen KH, Jakobsen E, Schousboe A, Waagepetersen HS, Rosenberg PA, Aldana BI (2021) Glutamate metabolism and recycling at the excitatory synapse in health and neurodegeneration. Neuropharmacology 196:108719. 10.1016/j.neuropharm.2021.108719 34273389 10.1016/j.neuropharm.2021.108719

[56] Hyder F, Fulbright RK, Shulman RG, Rothman DL (2013) Glutamatergic function in the resting awake human brain is supported by uniformly high oxidative energy. J Cereb Blood Flow Metab 33(3):339–347. 10.1038/jcbfm.2012.207 23299240 10.1038/jcbfm.2012.207 PMC3587823

[57] Rae CD, Baur JA, Borges K, Dienel G, Díaz-García CM, Douglass SR, Drew K, Duarte JMN, Duran J, Kann O, Kristian T, Lee-Liu D, Lindquist BE, McNay EC, Robinson MB, Rothman DL, Rowlands BD, Ryan TA, Scafidi J, Scafidi S, Shuttleworth CW, Swanson RA, Uruk G, Vardjan N, Zorec R, McKenna MC (2024) Brain energy metabolism: a roadmap for future research. J Neurochem 168(5):910–954. 10.1111/jnc.16032 38183680 10.1111/jnc.16032 PMC11102343

[58] Sibson NR, Dhankhar A, Mason GF, Rothman DL, Behar KL, Shulman RG (1998) Stoichiometric coupling of brain glucose metabolism and glutamatergic neuronal activity. Proc Natl Acad Sci U S A 95(1):316–321. 10.1073/pnas.95.1.316 9419373 10.1073/pnas.95.1.316 PMC18211

[59] Rothman DL, Dienel GA, Behar KL, Hyder F, DiNuzzo M, Giove F, Mangia S (2022) Glucose sparing by glycogenolysis (GSG) determines the relationship between brain metabolism and neurotransmission. J Cereb Blood Flow Metab 42(5):844–860. 10.1177/0271678X211064399 34994222 10.1177/0271678X211064399 PMC9254033

[60] Rose EM, Koo JC, Antflick JE, Ahmed SM, Angers S, Hampson DR (2009) Glutamate transporter coupling to Na,K-ATPase. J Neurosci 29(25):8143–8155. 10.1523/JNEUROSCI.1081-09.2009 19553454 10.1523/JNEUROSCI.1081-09.2009 PMC6666056

[61] Genda EN, Jackson JG, Sheldon AL, Locke SF, Greco TM, O’Donnell JC, Spruce LA, Xiao R, Guo W, Putt M, Seeholzer S, Ischiropoulos H, Robinson MB (2011) Co-compartmentalization of the astroglial glutamate transporter, GLT-1, with glycolytic enzymes and mitochondria. J Neurosci 31(50):18275–18288. 10.1523/JNEUROSCI.3305-11.2011 22171032 10.1523/JNEUROSCI.3305-11.2011 PMC3259858

[62] Bauer DE, Jackson JG, Genda EN, Montoya MM, Yudkoff M, Robinson MB (2012) The glutamate transporter, GLAST, participates in a macromolecular complex that supports glutamate metabolism. Neurochem Int 61(4):566–574. 10.1016/j.neuint.2012.01.013 22306776 10.1016/j.neuint.2012.01.013 PMC3350823

[63] Jackson JG, O’Donnell JC, Takano H, Coulter DA, Robinson MB (2014) Neuronal activity and glutamate uptake decrease mitochondrial mobility in astrocytes and position mitochondria near glutamate transporters. J Neurosci 34(5):1613–1624. 10.1523/JNEUROSCI.3510-13.2014 24478345 10.1523/JNEUROSCI.3510-13.2014 PMC3905137

[64] Ugbode CI, Hirst WD, Rattray M (2014) Neuronal influences are necessary to produce mitochondrial co-localization with glutamate transporters in astrocytes. J Neurochem 130(5):668–677. 10.1111/jnc.12759 24814819 10.1111/jnc.12759 PMC4283053

[65] Oz G, Berkich DA, Henry PG, Xu Y, LaNoue K, Hutson SM, Gruetter R (2004) Neuroglial metabolism in the awake rat brain: CO2 fixation increases with brain activity. J Neurosci 24(50):11273–11279. 10.1523/JNEUROSCI.3564-04.2004 15601933 10.1523/JNEUROSCI.3564-04.2004 PMC6730363

[66] Shen J, Petersen KF, Behar KL, Brown P, Nixon TW, Mason GF, Petroff OA, Shulman GI, Shulman RG, Rothman DL (1999) Determination of the rate of the glutamate/glutamine cycle in the human brain by in vivo 13 C NMR. Proc Natl Acad Sci U S A 96(14):8235–8240. 10.1073/pnas.96.14.8235 10393978 10.1073/pnas.96.14.8235 PMC22218

[67] Jeffrey FM, Marin-Valencia I, Good LB, Shestov AA, Henry PG, Pascual JM, Malloy CR (2013) Modeling of brain metabolism and pyruvate compartmentation using (13)C NMR in vivo: caution required. J Cereb Blood Flow Metab 33(8):1160–1167. 10.1038/jcbfm.2013.67 23652627 10.1038/jcbfm.2013.67 PMC3734769

[68] Sonnay S, Duarte JMN, Just N, Gruetter R (2017) Energy metabolism in the rat cortex under thiopental anaesthesia measured in vivo by 13 C MRS. J Neurosci Res 95(11):2297–2306. 10.1002/jnr.24032 28316083 10.1002/jnr.24032

[69] Girault FM, Sonnay S, Gruetter R, Duarte JMN (2019) Alterations of Brain Energy metabolism in type 2 Diabetic Goto-Kakizaki rats measured in vivo by 13 C magnetic resonance spectroscopy. Neurotox Res 36(2):268–278. 10.1007/s12640-017-9821-y 28971314 10.1007/s12640-017-9821-y

[70] Choi IY, Lei H, Gruetter R (2002) Effect of deep pentobarbital anesthesia on neurotransmitter metabolism in vivo: on the correlation of total glucose consumption with glutamatergic action. J Cereb Blood Flow Metab 22(11):1343–1351. 10.1097/01.WCB.0000040945.89393.46 12439292 10.1097/01.WCB.0000040945.89393.46

[71] Lai M, Lanz B, Poitry-Yamate C, Romero JF, Berset CM, Cudalbu C, Gruetter R (2018) In vivo 13 C MRS in the mouse brain at 14.1 Tesla and metabolic flux quantification under infusion of [1,6-13C2]glucose. J Cereb Blood Flow Metab 38(10):1701–1714. 10.1177/0271678X17734101 29047296 10.1177/0271678X17734101 PMC6168901

[72] Lebon V, Petersen KF, Cline GW, Shen J, Mason GF, Dufour S, Behar KL, Shulman GI, Rothman DL (2002) Astroglial contribution to brain energy metabolism in humans revealed by 13 C nuclear magnetic resonance spectroscopy: elucidation of the dominant pathway for neurotransmitter glutamate repletion and measurement of astrocytic oxidative metabolism. J Neurosci 22(5):1523–1531. 10.1523/JNEUROSCI.22-05-01523.2002 11880482 10.1523/JNEUROSCI.22-05-01523.2002 PMC2995528

[73] Mason GF, Petersen KF, de Graaf RA, Shulman GI, Rothman DL (2007) Measurements of the anaplerotic rate in the human cerebral cortex using 13 C magnetic resonance spectroscopy and [1-13 C] and [2-13 C] glucose. J Neurochem 100(1):73–86. 10.1111/j.1471-4159.2006.04200.x 17076763 10.1111/j.1471-4159.2006.04200.x PMC2995551

[74] Boumezbeur F, Mason GF, de Graaf RA, Behar KL, Cline GW, Shulman GI, Rothman DL, Petersen KF (2010) Altered brain mitochondrial metabolism in healthy aging as assessed by in vivo magnetic resonance spectroscopy. J Cereb Blood Flow Metab 30(1):211–221. 10.1038/jcbfm.2009.197 19794401 10.1038/jcbfm.2009.197 PMC2949111

[75] Jiang L, Gulanski BI, De Feyter HM, Weinzimer SA, Pittman B, Guidone E, Koretski J, Harman S, Petrakis IL, Krystal JH, Mason GF (2013) Increased brain uptake and oxidation of acetate in heavy drinkers. J Clin Invest 123(4):1605–1614. 10.1172/JCI65153 23478412 10.1172/JCI65153 PMC3613911

[76] de Graaf RA, Mason GF, Patel AB, Rothman DL, Behar KL (2004) Regional glucose metabolism and glutamatergic neurotransmission in rat brain in vivo. Proc Natl Acad Sci U S A 101(34):12700–12705. 10.1073/pnas.0405065101 15310848 10.1073/pnas.0405065101 PMC515118

[77] Patel AB, de Graaf RA, Mason GF, Kanamatsu T, Rothman DL, Shulman RG, Behar KL (2004) Glutamatergic neurotransmission and neuronal glucose oxidation are coupled during intense neuronal activation. J Cereb Blood Flow Metab 24(9):972–985. 10.1097/01.WCB.0000126234.16188.71 15356418 10.1097/01.WCB.0000126234.16188.71

[78] van Eijsden P, Behar KL, Mason GF, Braun KP, de Graaf RA (2010) In vivo neurochemical profiling of rat brain by 1H-[13 C] NMR spectroscopy: cerebral energetics and glutamatergic/GABAergic neurotransmission. J Neurochem 112(1):24–33. 10.1111/j.1471-4159.2009.06428.x 19818103 10.1111/j.1471-4159.2009.06428.x PMC2843425

[79] Lin AL, Coman D, Jiang L, Rothman DL, Hyder F (2014) Caloric restriction impedes age-related decline of mitochondrial function and neuronal activity. J Cereb Blood Flow Metab 34(9):1440–1443. 10.1038/jcbfm.2014.114 24984898 10.1038/jcbfm.2014.114 PMC4158670

[80] Patel AB, de Graaf RA, Rothman DL, Behar KL (2015) Effects of γ-Aminobutyric acid transporter 1 inhibition by tiagabine on brain glutamate and γ-Aminobutyric acid metabolism in the anesthetized rat in vivo. J Neurosci Res 93(7):1101–1108. 10.1002/jnr.23548 25663257 10.1002/jnr.23548 PMC4441585

[81] Tiwari V, Ambadipudi S, Patel AB (2013) Glutamatergic and GABAergic TCA cycle and neurotransmitter cycling fluxes in different regions of mouse brain. J Cereb Blood Flow Metab 33(10):1523–1531. 10.1038/jcbfm.2013.114 23838829 10.1038/jcbfm.2013.114 PMC3790929

[82] Mekle R, Mlynárik V, Gambarota G, Hergt M, Krueger G, Gruetter R (2009) MR spectroscopy of the human brain with enhanced signal intensity at ultrashort echo times on a clinical platform at 3T and 7T. Magn Reson Med 61(6):1279–1285. 10.1002/mrm.21961 19319893 10.1002/mrm.21961

[83] Marjańska M, Auerbach EJ, Valabrègue R, Van de Moortele PF, Adriany G, Garwood M (2012) Localized 1H NMR spectroscopy in different regions of human brain in vivo at 7 T: T2 relaxation times and concentrations of cerebral metabolites. NMR Biomed 25(2):332–339. 10.1002/nbm.1754 21796710 10.1002/nbm.1754 PMC3357544

[84] Marjańska M, McCarten JR, Hodges J, Hemmy LS, Grant A, Deelchand DK, Terpstra M (2017) Region-specific aging of the human brain as evidenced by neurochemical profiles measured noninvasively in the posterior cingulate cortex and the occipital lobe using 1H magnetic resonance spectroscopy at 7 T. Neuroscience 354:168–177. 10.1016/j.neuroscience.2017.04.035 28476320 10.1016/j.neuroscience.2017.04.035 PMC5516630

[85] Deelchand DK, Van de Moortele PF, Adriany G, Iltis I, Andersen P, Strupp JP, Vaughan JT, Uğurbil K, Henry PG (2010) In vivo 1H NMR spectroscopy of the human brain at 9.4 T: initial results. J Magn Reson 206(1):74–80. 10.1016/j.jmr.2010.06.006 20598925 10.1016/j.jmr.2010.06.006 PMC2940249

[86] Gambarota G, Mekle R, Xin L, Hergt M, van der Zwaag W, Krueger G, Gruetter R (2009) In vivo measurement of glycine with short echo-time 1H MRS in human brain at 7 T. MAGMA 22(1):1–4. 10.1007/s10334-008-0152-0 18949497 10.1007/s10334-008-0152-0

[87] Wang WT, Lee P, Dong Y, Yeh HW, Kim J, Weiner CP, Brooks WM, Choi IY (2016) In vivo neurochemical characterization of developing Guinea pigs and the effect of chronic fetal hypoxia. Neurochem Res 41(7):1831–1843. 10.1007/s11064-016-1924-y 27233245 10.1007/s11064-016-1924-y

[88] Lei H, Duarte JM, Mlynarik V, Python A, Gruetter R (2010) Deep thiopental anesthesia alters steady-state glucose homeostasis but not the neurochemical profile of rat cortex. J Neurosci Res 88(2):413–419. 10.1002/jnr.22212 19746430 10.1002/jnr.22212

[89] Duarte JMN, Skoug C, Silva HB, Carvalho RA, Gruetter R, Cunha RA (2019) Impact of Caffeine Consumption on Type 2 Diabetes-Induced spatial memory impairment and neurochemical alterations in the Hippocampus. Front Neurosci 12:1015. 10.3389/fnins.2018.01015 30686981 10.3389/fnins.2018.01015 PMC6333904

[90] Xin L, Gambarota G, Duarte JM, Mlynárik V, Gruetter R (2010) Direct in vivo measurement of glycine and the neurochemical profile in the rat medulla oblongata. NMR Biomed 23(9):1097–1102. 10.1002/nbm.1537 20963803 10.1002/nbm.1537

[91] Harris JL, Yeh HW, Swerdlow RH, Choi IY, Lee P, Brooks WM (2014) High-field proton magnetic resonance spectroscopy reveals metabolic effects of normal brain aging. Neurobiol Aging 35(7):1686–1694. 10.1016/j.neurobiolaging.2014.01.018 24559659 10.1016/j.neurobiolaging.2014.01.018 PMC4174428

[92] Cuellar-Baena S, Landeck N, Sonnay S, Buck K, Mlynarik V, In ‘t Zandt R, Kirik D (2016) Assessment of brain metabolite correlates of adeno-associated virus-mediated over-expression of human alpha-synuclein in cortical neurons by in vivo (1) H-MR spectroscopy at 9.4 T. J Neurochem 137(5):806–819. 10.1111/jnc.13547 26811128 10.1111/jnc.13547

[93] Lanzillotta C, Tramutola A, Lanzillotta S, Greco V, Pagnotta S, Sanchini C, Di Angelantonio S, Forte E, Rinaldo S, Paone A, Cutruzzolà F, Cimini FA, Barchetta I, Cavallo MG, Urbani A, Butterfield DA, Di Domenico F, Paul BD, Perluigi M, Duarte JMN, Barone E (2024) Biliverdin Reductase-A integrates insulin signaling with mitochondrial metabolism through phosphorylation of GSK3β. Redox Biol 73:103221. 10.1016/j.redox.2024.103221 38843768 10.1016/j.redox.2024.103221 PMC11190564

[94] Cherix A, Larrieu T, Grosse J, Rodrigues J, McEwen B, Nasca C, Gruetter R, Sandi C (2020) Metabolic signature in nucleus accumbens for anti-depressant-like effects of acetyl-L-carnitine. Elife 9:e50631. 10.7554/eLife.50631PMID: 31922486; PMCID: PMC6970538 31922486 10.7554/eLife.50631 PMC6970538

[95] Larrieu T, Cherix A, Duque A, Rodrigues J, Lei H, Gruetter R, Sandi C (2017) Hierarchical Status predicts behavioral vulnerability and nucleus Accumbens Metabolic Profile following chronic social defeat stress. Curr Biol 27(14):2202–2210e4. 10.1016/j.cub.2017.06.027Epub 2017 Jul 14. PMID: 28712571 28712571 10.1016/j.cub.2017.06.027

[96] Kim J, Choi IY, Duff KE, Lee P (2017) Progressive pathological changes in Neurochemical Profile of the Hippocampus and early changes in the olfactory bulbs of tau transgenic mice (rTg4510). Neurochem Res 42(6):1649–1660. 10.1007/s11064-017-2298-5 28523532 10.1007/s11064-017-2298-5 PMC5565734

[97] Lizarbe B, Soares AF, Larsson S, Duarte JMN (2019) Neurochemical modifications in the Hippocampus, Cortex and Hypothalamus of mice exposed to Long-Term High-Fat Diet. Front Neurosci 12:985. 10.3389/fnins.2018.00985 30670942 10.3389/fnins.2018.00985 PMC6331468

[98] Lei H, Berthet C, Hirt L, Gruetter R (2009) Evolution of the neurochemical profile after transient focal cerebral ischemia in the mouse brain. J Cereb Blood Flow Metab 29(4):811–819. 10.1038/jcbfm.2009.8 19223915 10.1038/jcbfm.2009.8

[99] Garcia-Serrano AM, Mohr AA, Philippe J, Skoug C, Spégel P, Duarte JMN (2022) Cognitive impairment and Metabolite Profile alterations in the Hippocampus and Cortex of male and female mice exposed to a Fat and Sugar-Rich Diet are normalized by Diet reversal. Aging Dis 13(1):267–283. 10.14336/AD.2021.0720 35111373 10.14336/AD.2021.0720 PMC8782561

[100] Garcia-Serrano AM, Vieira JPP, Fleischhart V, Duarte JMN (2023) Taurine and N-acetylcysteine treatments prevent memory impairment and metabolite profile alterations in the hippocampus of high-fat diet-fed female mice. Nutr Neurosci 26(11):1090–1102. 10.1080/1028415X.2022.2131062 36222315 10.1080/1028415X.2022.2131062

[101] Kulak A, Duarte JM, Do KQ, Gruetter R (2010) Neurochemical profile of the developing mouse cortex determined by in vivo 1H NMR spectroscopy at 14.1 T and the effect of recurrent anaesthesia. J Neurochem 115(6):1466–1477. 10.1111/j.1471-4159.2010.07051.x 20946416 10.1111/j.1471-4159.2010.07051.x

[102] Duarte JMN, Kulak A, Gholam-Razaee MM, Cuenod M, Gruetter R, Do KQ (2012) N-acetylcysteine normalizes neurochemical changes in the glutathione-deficient schizophrenia mouse model during development. Biol Psychiatry 71(11):1006–1014. 10.1016/j.biopsych.2011.07.035 21945305 10.1016/j.biopsych.2011.07.035

[103] Duarte JM, Do KQ, Gruetter R (2014) Longitudinal neurochemical modifications in the aging mouse brain measured in vivo by 1H magnetic resonance spectroscopy. Neurobiol Aging 35(7):1660–1668. 10.1016/j.neurobiolaging.2014.01.135 24560998 10.1016/j.neurobiolaging.2014.01.135

[104] Corcoba A, Steullet P, Duarte JM, Van de Looij Y, Monin A, Cuenod M, Gruetter R, Do KQ (2015) Glutathione deficit affects the Integrity and function of the Fimbria/Fornix and Anterior Commissure in mice: relevance for Schizophrenia. Int J Neuropsychopharmacol 19(3):pyv110. 10.1093/ijnp/pyv110 26433393 10.1093/ijnp/pyv110 PMC4815475

[105] Gapp K, Corcoba A, van Steenwyk G, Mansuy IM, Duarte JM (2017) Brain metabolic alterations in mice subjected to postnatal traumatic stress and in their offspring. J Cereb Blood Flow Metab 37(7):2423–2432. 10.1177/0271678X16667525 27604311 10.1177/0271678X16667525 PMC5531341

[106] Tkác I, Henry PG, Andersen P, Keene CD, Low WC, Gruetter R (2004) Highly resolved in vivo 1H NMR spectroscopy of the mouse brain at 9.4 T. Magn Reson Med 52(3):478–484. 10.1002/mrm.20184 15334565 10.1002/mrm.20184

[107] Tkáč I, Xie T, Shah N, Larson S, Dubinsky JM, Gomez-Pastor R, McLoughlin HS, Orr HT, Eberly LE, Öz G (2023) Regional sex differences in neurochemical profiles of healthy mice measured by magnetic resonance spectroscopy at 9.4 tesla. Front Neurosci 17:1278828. 10.3389/fnins.2023.1278828 37954878 10.3389/fnins.2023.1278828 PMC10634209

[108] Mohr AA, Garcia-Serrano AM, Vieira JP, Skoug C, Davidsson H, Duarte JM (2021) A glucose-stimulated BOLD fMRI study of hypothalamic dysfunction in mice fed a high-fat and high-sucrose diet. J Cereb Blood Flow Metab 41(7):1734–1743. 10.1177/0271678X20942397 32757742 10.1177/0271678X20942397 PMC8217889

[109] Thaler JP, Yi CX, Schur EA, Guyenet SJ, Hwang BH, Dietrich MO, Zhao X, Sarruf DA, Izgur V, Maravilla KR, Nguyen HT, Fischer JD, Matsen ME, Wisse BE, Morton GJ, Horvath TL, Baskin DG, Tschöp MH, Schwartz MW (2012) Obesity is associated with hypothalamic injury in rodents and humans. J Clin Invest 122(1):153–162. 10.1172/JCI59660 22201683 10.1172/JCI59660 PMC3248304

[110] Ardenkjaer-Larsen JH, Fridlund B, Gram A, Hansson G, Hansson L, Lerche MH, Servin R, Thaning M, Golman K (2003) Increase in signal-to-noise ratio of > 10,000 times in liquid-state NMR. Proc Natl Acad Sci U S A 100(18):10158–10163. 10.1073/pnas.1733835100 12930897 10.1073/pnas.1733835100 PMC193532

[111] Mishkovsky M, Anderson B, Karlsson M, Lerche MH, Sherry AD, Gruetter R, Kovacs Z, Comment A (2017) Measuring glucose cerebral metabolism in the healthy mouse using hyperpolarized 13 C magnetic resonance. Sci Rep 7(1):11719. 10.1038/s41598-017-12086-z 28916775 10.1038/s41598-017-12086-z PMC5601924

[112] Takado Y, Cheng T, Bastiaansen JAM, Yoshihara HAI, Lanz B, Mishkovsky M, Lengacher S, Comment A (2018) Hyperpolarized 13 C magnetic resonance spectroscopy reveals the rate-limiting role of the blood-brain barrier in the cerebral uptake and metabolism of l-Lactate in vivo. ACS Chem Neurosci 9(11):2554–2562. 10.1021/acschemneuro.8b00066 29771492 10.1021/acschemneuro.8b00066 PMC6119468

[113] Bøgh N, Grist JT, Rasmussen CW, Bertelsen LB, Hansen ESS, Blicher JU, Tyler DJ, Laustsen C (2022) Lactate saturation limits bicarbonate detection in hyperpolarized 13 C-pyruvate MRI of the brain. Magn Reson Med 88(3):1170–1179. 10.1002/mrm.29290 35533254 10.1002/mrm.29290 PMC9322338

[114] Hurd RE, Yen YF, Tropp J, Pfefferbaum A, Spielman DM, Mayer D (2010) Cerebral dynamics and metabolism of hyperpolarized [1-(13)C]pyruvate using time-resolved MR spectroscopic imaging. J Cereb Blood Flow Metab 30(10):1734–1741. 10.1038/jcbfm.2010.93 20588318 10.1038/jcbfm.2010.93 PMC2975615

[115] Eichhorn TR, Takado Y, Salameh N, Capozzi A, Cheng T, Hyacinthe JN, Mishkovsky M, Roussel C, Comment A (2013) Hyperpolarization without persistent radicals for in vivo real-time metabolic imaging. Proc Natl Acad Sci U S A 110(45):18064–18069. 10.1073/pnas.1314928110 24145405 10.1073/pnas.1314928110 PMC3831441

[116] Park JM, Josan S, Grafendorfer T, Yen YF, Hurd RE, Spielman DM, Mayer D (2013) Measuring mitochondrial metabolism in rat brain in vivo using MR Spectroscopy of hyperpolarized [2-¹³C]pyruvate. NMR Biomed 26(10):1197–1203. 10.1002/nbm.2935 23553852 10.1002/nbm.2935 PMC3726546

[117] Flatt E, Lanz B, Pilloud Y, Capozzi A, Lerche MH, Gruetter R, Mishkovsky M (2021) Measuring glycolytic activity with hyperpolarized [2H7, U-13C6] D-Glucose in the naive mouse brain under different anesthetic conditions. Metabolites 11(7):413. 10.3390/metabo11070413 34201777 10.3390/metabo11070413 PMC8303162

[118] Boretius S, Tammer R, Michaelis T, Brockmöller J, Frahm J (2013) Halogenated volatile anesthetics alter brain metabolism as revealed by Proton magnetic resonance spectroscopy of mice in vivo. NeuroImage 69:244–255. 10.1016/j.neuroimage.2012.12.020 23266699 10.1016/j.neuroimage.2012.12.020

[119] Mishkovsky M, Comment A, Gruetter R (2012) In vivo detection of brain Krebs cycle intermediate by hyperpolarized magnetic resonance. J Cereb Blood Flow Metab 32(12):2108–2113. 10.1038/jcbfm.2012.136 22990416 10.1038/jcbfm.2012.136 PMC3519415

[120] Lu M, Zhu XH, Zhang Y, Mateescu G, Chen W (2017) Quantitative assessment of brain glucose metabolic rates using in vivo deuterium magnetic resonance spectroscopy. J Cereb Blood Flow Metab 37(11):3518–3530. 10.1177/0271678X17706444 28503999 10.1177/0271678X17706444 PMC5669347

[121] De Feyter HM, Behar KL, Corbin ZA, Fulbright RK, Brown PB, McIntyre S, Nixon TW, Rothman DL, de Graaf RA (2018) Deuterium metabolic imaging (DMI) for MRI-based 3D mapping of metabolism in vivo. Sci Adv 4(8):eaat7314. 10.1126/sciadv.aat7314 30140744 10.1126/sciadv.aat7314 PMC6105304

[122] Soni ND, Swain A, Jacobs P, Juul H, Armbruster R, Nanga RPR, Nath K, Wiers C, Detre J, Reddy R (2023) In vivo assessment of β-hydroxybutyrate metabolism in mouse brain using deuterium (^2^H) MRS. Magn Reson Med 90(1):259–269. 10.1002/mrm.29648 36971349 10.1002/mrm.29648 PMC10662955

[123] Rich LJ, Bagga P, Wilson NE, Schnall MD, Detre JA, Haris M, Reddy R (2020) 1H magnetic resonance spectroscopy of 2H-to-1H exchange quantifies the dynamics of cellular metabolism in vivo. Nat Biomed Eng 4(3):335–342. 10.1038/s41551-019-0499-8 31988460 10.1038/s41551-019-0499-8 PMC7071956

[124] Bednarik P, Goranovic D, Svatkova A, Niess F, Hingerl L, Strasser B, Deelchand DK, Spurny-Dworak B, Krssak M, Trattnig S, Hangel G, Scherer T, Lanzenberger R, Bogner W (2023) 1H magnetic resonance spectroscopic imaging of deuterated glucose and of neurotransmitter metabolism at 7 T in the human brain. Nat Biomed Eng 7(8):1001–1013. 10.1038/s41551-023-01035-z 37106154 10.1038/s41551-023-01035-z PMC10861140

[125] Pasanta D, He JL, Ford T, Oeltzschner G, Lythgoe DJ, Puts NA (2023) Functional MRS studies of GABA and glutamate/Glx - A systematic review and meta-analysis. Neurosci Biobehav Rev 144:104940. 10.1016/j.neubiorev.2022.104940 36332780 10.1016/j.neubiorev.2022.104940 PMC9846867

[126] Wilson R, Thomas A, Mayhew SD (2020) Spatially congruent negative BOLD responses to different stimuli do not summate in visual cortex. Neuroimage.;218:116891. 10.1016/j.neuroimage.2020.116891. Epub 2020 May 11. PMID: 32438052 10.1016/j.neuroimage.2020.116891 32438052

[127] Shmuel A, Yacoub E, Pfeuffer J, Van de Moortele PF, Adriany G, Hu X, Ugurbil K (2002) Sustained negative BOLD, blood flow and oxygen consumption response and its coupling to the positive response in the human brain. Neuron.;36(6):1195– 210. 10.1016/s0896-6273(02)01061-9. PMID: 12495632 10.1016/s0896-6273(02)01061-9 12495632

[128] Walter SA, Forsgren M, Lundengård K, Simon R, Torkildsen Nilsson M, Söderfeldt B, Lundberg P, Engström M (2016) Positive Allosteric Modulator of GABA lowers BOLD responses in the Cingulate Cortex. PLoS ONE 11(3):e0148737. 10.1371/journal.pone.0148737PMID: 26930498; PMCID: PMC4773017 26930498 10.1371/journal.pone.0148737 PMC4773017

[129] Seuwen A, Schroeter A, Grandjean J, Schlegel F, Rudin M (2019) Functional spectroscopic imaging reveals specificity of glutamate response in mouse brain to peripheral sensory stimulation. Sci Rep 9(1):10563. 10.1038/s41598-019-46477-1 31332260 10.1038/s41598-019-46477-1 PMC6646328

[130] Reimann HM, Niendorf T (2020) The (Un)conscious mouse as a model for human brain functions: Key principles of Anesthesia and their impact on translational neuroimaging. Front Syst Neurosci 14:8. 10.3389/fnsys.2020.00008 32508601 10.3389/fnsys.2020.00008 PMC7248373

[131] Takado Y, Takuwa H, Sampei K, Urushihata T, Takahashi M, Shimojo M, Uchida S, Nitta N, Shibata S, Nagashima K, Ochi Y, Ono M, Maeda J, Tomita Y, Sahara N, Near J, Aoki I, Shibata K, Higuchi M (2022) MRS-measured glutamate versus GABA reflects excitatory versus inhibitory neural activities in awake mice. J Cereb Blood Flow Metab 42(1):197–212. 10.1177/0271678X211045449 34515548 10.1177/0271678X211045449 PMC8721779

[132] Rothman DL, Petroff OA, Behar KL, Mattson RH (1993) Localized 1H NMR measurements of gamma-aminobutyric acid in human brain in vivo. Proc Natl Acad Sci USA 90(12):5662–5666. 10.1073/pnas.90.12.5662 8516315 10.1073/pnas.90.12.5662 PMC46781

[133] Mescher M, Merkle H, Kirsch J, Garwood M, Gruetter R (1998) Simultaneous in vivo spectral editing and water suppression. NMR Biomed 11(6):266–272. 10.1002/(sici)1099-1492(199810)11:6-266::aid-nbm530-3.0.co;2-j 9802468 10.1002/(sici)1099-1492(199810)11:6<266::aid-nbm530>3.0.co;2-j

[134] Kapogiannis D, Reiter DA, Willette AA, Mattson MP (2013) Posteromedial cortex glutamate and GABA predict intrinsic functional connectivity of the default mode network. NeuroImage 64:112–119. 10.1016/j.neuroimage.2012.09.029 23000786 10.1016/j.neuroimage.2012.09.029 PMC3801193

[135] Stagg CJ, Bachtiar V, Amadi U, Gudberg CA, Ilie AS, Sampaio-Baptista C, O’Shea J, Woolrich M, Smith SM, Filippini N, Near J, Johansen-Berg H (2014) Local GABA concentration is related to network-level resting functional connectivity. Elife 3:e01465 24668166 10.7554/eLife.01465 PMC3964822

[136] Duncan NW, Wiebking C, Northoff G (2014) Associations of regional GABA and glutamate with intrinsic and extrinsic neural activity in humans - a review of multimodal imaging studies. Neurosci Biobehav Rev 47:36–52. 10.1016/j.neubiorev.2014.07.016 25066091 10.1016/j.neubiorev.2014.07.016

[137] Taylor MK, Swerdlow RH, Sullivan DK (2019) Dietary neuroketotherapeutics for Alzheimer’s Disease: an evidence update and the potential role for Diet Quality. Nutrients 11(8):1910. 10.3390/nu11081910 31443216 10.3390/nu11081910 PMC6722814

[138] Myette-Côté É, Soto-Mota A, Cunnane SC (2022) Ketones: potential to achieve brain energy rescue and sustain cognitive health during ageing. Br J Nutr 128(3):407–423. 10.1017/S0007114521003883 34581265 10.1017/S0007114521003883

[139] van Gemert LA, de Galan BE, Wevers RA, Ter Heine R, Willemsen MA (2022) Lactate infusion as therapeutical intervention: a scoping review. Eur J Pediatr 181(6):2227–2235. 10.1007/s00431-022-04446-3 35304646 10.1007/s00431-022-04446-3 PMC9110504

[140] Duarte JMN (2024) Concentrations of glutamate and N-acetylaspartate detected by magnetic resonance spectroscopy in the rat hippocampus correlate with hippocampal-dependent spatial memory performance. Front Mol Neurosci 17:1458070. 10.3389/fnmol.2024.1458070 39219740 10.3389/fnmol.2024.1458070 PMC11362093

[141] Koolschijn RS, Shpektor A, Clarke WT, Ip IB, Dupret D, Emir UE, Barron HC (2021) Memory recall involves a transient break in excitatory-inhibitory balance. Elife 10:e70071. 10.7554/eLife.70071 34622779 10.7554/eLife.70071 PMC8516417

[142] van Opstal AM, Westerink AM, Teeuwisse WM, van der Geest MA, van Furth EF, van der Grond J (2015) Hypothalamic BOLD response to glucose intake and hypothalamic volume are similar in anorexia nervosa and healthy control subjects. Front Neurosci 9:159. 10.3389/fnins.2015.00159 25999808 10.3389/fnins.2015.00159 PMC4419717

[143] Schridde U, Khubchandani M, Motelow JE, Sanganahalli BG, Hyder F, Blumenfeld H (2008) Negative BOLD with large increases in neuronal activity. Cereb Cortex 18(8):1814–1827. 10.1093/cercor/bhm208 18063563 10.1093/cercor/bhm208 PMC2790390

[144] Arnold PK, Finley LWS (2023) Regulation and function of the mammalian tricarboxylic acid cycle. J Biol Chem 299(2):102838. 10.1016/j.jbc.2022.102838 36581208 10.1016/j.jbc.2022.102838 PMC9871338

[145] Bosier B, Bellocchio L, Metna-Laurent M, Soria-Gomez E, Matias I, Hebert-Chatelain E, Cannich A, Maitre M, Leste-Lasserre T, Cardinal P, Mendizabal-Zubiaga J, Canduela MJ, Reguero L, Hermans E, Grandes P, Cota D, Marsicano G (2013) Astroglial CB1 cannabinoid receptors regulate leptin signaling in mouse brain astrocytes. Mol Metab 2(4):393–404. 10.1016/j.molmet.2013.08.001 24327955 10.1016/j.molmet.2013.08.001 PMC3854987

[146] Köfalvi A, Lemos C, Martín-Moreno AM, Pinheiro BS, García-García L, Pozo MA, Valério-Fernandes Â, Beleza RO, Agostinho P, Rodrigues RJ, Pasquaré SJ, Cunha RA, de Ceballos ML (2016) Stimulation of brain glucose uptake by cannabinoid CB2 receptors and its therapeutic potential in Alzheimer’s disease. Neuropharmacology 110(Pt A):519–529. 10.1016/j.neuropharm.2016.03.015 26976670 10.1016/j.neuropharm.2016.03.015

[147] Allaman I, Lengacher S, Magistretti PJ, Pellerin L (2003) A2B receptor activation promotes glycogen synthesis in astrocytes through modulation of gene expression. Am J Physiol Cell Physiol 284(3):C696–704. 10.1152/ajpcell.00202.2002 12421692 10.1152/ajpcell.00202.2002

[148] Theparambil SM, Kopach O, Braga A, Nizari S, Hosford PS, Sagi-Kiss V, Hadjihambi A, Konstantinou C, Esteras N, Del Gutierrez A, Ackland GL, Teschemacher AG, Dale N, Eckle T, Andrikopoulos P, Rusakov DA, Kasparov S, Gourine AV (2024) Adenosine signalling to astrocytes coordinates brain metabolism and function. Nature 632(8023):139–146. 10.1038/s41586-024-07611-w 38961289 10.1038/s41586-024-07611-w PMC11291286

[149] Barros LF, Bolaños JP, Bonvento G, Bouzier-Sore AK, Brown A, Hirrlinger J, Kasparov S, Kirchhoff F, Murphy AN, Pellerin L, Robinson MB, Weber B (2018) Current technical approaches to brain energy metabolism. Glia 66(6):1138–1159. 10.1002/glia.23248 29110344 10.1002/glia.23248 PMC5903992

[150] Ferenczi EA, Zalocusky KA, Liston C, Grosenick L, Warden MR, Amatya D, Katovich K, Mehta H, Patenaude B, Ramakrishnan C, Kalanithi P, Etkin A, Knutson B, Glover GH, Deisseroth K (2016) Prefrontal cortical regulation of brainwide circuit dynamics and reward-related behavior. Science 351(6268):aac9698. 10.1126/science.aac9698 26722001 10.1126/science.aac9698 PMC4772156

[151] Takata N, Sugiura Y, Yoshida K, Koizumi M, Hiroshi N, Honda K, Yano R, Komaki Y, Matsui K, Suematsu M, Mimura M, Okano H, Tanaka KF (2018) Optogenetic astrocyte activation evokes BOLD fMRI response with oxygen consumption without neuronal activity modulation. Glia 66(9):2013–2023. 10.1002/glia.23454 29845643 10.1002/glia.23454

[152] Just N, Faber C (2019) Probing activation-induced neurochemical changes using optogenetics combined with functional magnetic resonance spectroscopy: a feasibility study in the rat primary somatosensory cortex. J Neurochem 150(4):402–419. 10.1111/jnc.14799 31222733 10.1111/jnc.14799

