# Supplementary material for: Challenges of Investigating Compartmentalized Brain Energy Metabolism Using Nuclear Magnetic Resonance Spectroscopy in vivo
Source: Neurochem Res. 2025 Jan 4;50(1):73. doi: 10.1007/s11064-024-04324-4 (PMC11700056; doi:10.1007/s11064-024-04324-4)
Supplement: Supplementary file 1 — Supplementary Material 1 [file 11064_2024_4324_MOESM1_ESM.pdf]

Table S1. Metabolic fluxes (in µmol/g/min) estimated from ^{13}C tracing experiments *in vivo* or *ex vivo* using mathematical models with 2 compartments (neurons and astroglia) or 3 compartments (GABAergic neurons, Glutamatergic neurons and astroglia). Fluxes in pink were calculated from reported values.

		^{13}C tracer	anesthesia	fixed V_{TCA} or V_{PC}	model and experimental conditions	brain area	V_{NT} **	V_{TCA}^{n} **	V_{TCA}^{g}	V_{PC}	$CMR_{glc(ox)}$ ***	V_{NT}^{GABA}	V_{NT}^{Glu}	V_{TCA}^{GABA}	V_{TCA}^{Glu}
**Human**	Shen *et al*. 1999 [115]	Glc_1		-		Cortex	0.32	0.71	0.06	0.04	0.41				
	Gruetter *et al*. 2001 [36]	Glc_1		-		Cortex	0.17	0.57	0.15	0.09	0.41				
	Lebon *et al*. 2002 [121]	Ac_2		$V_{TCA}^{n} = 0.80$, $V_{PC} = 0.04$		Cortex	0.32		0.14		0.49				
	Mason *et al*. 2007 [122] *	Glc_1 & Glc_2		$V_{TCA}^{n} = 0.73$, $V_{TCA}^{g} = 0.14$, $V_{gln} = 0.32$		Cortex	0.34	0.72		0.022	0.44				
	Boumezbeur *et al*. 2010 [123]	Glc_1 & Ac_2		$V_{PC} = 0.06*V_{NT}$	Young	Cortex	0.16	0.53	0.13	0.010	0.33				
	Boumezbeur *et al*. 2010 [123]	Glc_1 & Ac_2		$V_{PC} = 0.06*V_{NT}$	Elderly	Cortex	0.13	0.38	0.17	0.008	0.28				
	Jiang *et al*. 2013 [124]	Ac_2		$V_{TCA}^{n} = 0.73$, $V_{PC} = 0.02$		Cortex	0.18	0.73	0.15		0.45				
**Rat**	Choi *et al*. 2002 [119]	Glc_1	deep thiopental	-	Sprague Dawley rat	whole brain	0.04	0.35	0.29	0.03	0.34				
	Oz *et al*. 2004 [65]	Glc_{16}	none; awake	-	Sprague Dawley rat	whole brain	0.57	1.13	0.54	0.14	0.91				
	de Graaf *et al*. 2004 [125]	Glc_{16}	halothane	$V_{TCA}^{g} = 0.05*V_{TCA}^{n}$, $V_{PC} = 0.096$	Sprague Dawley rat	Cortex	0.28	0.73			0.43				
	de Graaf *et al*. 2004 [125]	Glc_{16}	halothane	$V_{TCA}^{g} = 0.05*V_{TCA}^{n}$, $V_{PC} = 0.096$	Sprague Dawley rat	Corpus calosum	0.14	0.42			0.27				
	de Graaf *et al*. 2004 [125]	Glc_{16}	halothane	$V_{TCA}^{g} = 0.05*V_{TCA}^{n}$, $V_{PC} = 0.096$	Sprague Dawley rat	subcortical area	0.19	0.44			0.28				
	Patel *et al*. 2004 [126]	Glc_{16}	halothane	$V_{TCA}^{g} = 0.15*(V_{TCA}^{n}+V_{TCA}^{g})$, $V_{PC} = 0.20*(V_{PC}+V_{NT})$	Wistar rat	Cortex	0.22	0.52			0.33				
	Patel *et al*. 2004 [126]	Glc_{16}	halothane	$V_{TCA}^{g} = 0.15*(V_{TCA}^{n}+V_{TCA}^{g})$, $V_{PC} = 0.20*(V_{PC}+V_{NT})$	Wistar rat with bicuculline-induced seizures	Cortex	0.52	1.14			0.74				
	Patel *et al*. 2005 [30]	Glc_{16} & Ac_2	deep pentobarbital	$V_{TCA}^{g} = 0.15*(V_{TCA}^{n}+V_{TCA}^{g})$, $V_{PC} = 0.20*(V_{PC}+V_{NT})$	Sprague Dawley rat	Cortex	0.015	0.35	0.06	0.004	0.21	<0.015	<0.015	0.060	0.29
	Patel *et al*. 2005 [30]	Glc_{16} & Ac_2	halothane	$V_{TCA}^{g} = 0.15*(V_{TCA}^{n}+V_{TCA}^{g})$, $V_{PC} = 0.20*(V_{PC}+V_{NT})$	Sprague Dawley rat	Cortex	0.60	1.20	0.21	0.151	0.78	0.107	0.50	0.214	0.99
	Chowdhury *et al*. 2007 [31]	Glc_{16} & Ac_2	urethane	$V_{TCA}^{g} = 0.15*(V_{TCA}^{n}+V_{TCA}^{g})$, $V_{PC} = 0.20*(V_{PC}+V_{NT})$	Sprague Dawley rat P30	Cortex	0.34	0.70	0.12	0.086	0.46	0.062	0.28	0.144	0.56
	Chowdhury *et al*. 2007 [31]	Glc_{16} & Ac_2	urethane	$V_{TCA}^{g} = 0.15*(V_{TCA}^{n}+V_{TCA}^{g})$, $V_{PC} = 0.20*(V_{PC}+V_{NT})$	Sprague Dawley rat P10	Cortex	0.089	0.24	0.04	0.022	0.15	0.009	0.08	0.029	0.21
	van Eijsden *et al*. 2010 [127]	Glc_U & Ac_2	halothane	$V_{PC} = 0.20*(V_{PC}+V_{NT})$	Wistar rat	Cortex+Hippocampus	0.31	0.53	0.14	0.077	0.37	0.033	0.27	0.062	0.47
	Duarte *et al*. 2011 [37]	Glc_{16}	α-Chloralose	-	Sprague Dawley rat	whole brain	0.11	0.45	0.28	0.069	0.40				
	Jeffrey *et al*. 2013 [116]	Glc_{16}	α-Chloralose	-	Sprague Dawley rat	whole brain	0.12	0.43	0.25	0.07	0.38				
	Duarte & Gruetter, 2013 [32]	Glc_{16}	α-Chloralose	-	Sprague Dawley rat	whole brain	0.24	0.40	0.44	0.092	0.47	0.053	0.18	0.070	0.33
	Lanz *et al*. 2014 [53]	Ac_2	α-Chloralose	-	Sprague Dawley rat	whole brain	0.15	0.37	0.36	0.087	0.41				
	Lin *et al*. 2014 [128]	Glc_{16}	α-chloralose	$V_{TCA}^{g} = 0.15*(V_{TCA}^{n}+V_{TCA}^{g})$, $V_{PC} = 0.20*(V_{PC}+V_{NT})$	Fischer 344 Brown-Norway F1 rat, young	Cortex	0.23	0.49	0.09	0.058	0.32				
	Lin *et al*. 2014 [128]	Glc_{16}	α-chloralose	$V_{TCA}^{g} = 0.15*(V_{TCA}^{n}+V_{TCA}^{g})$, $V_{PC} = 0.20*(V_{PC}+V_{NT})$	Fischer 344 Brown-Norway F1 rat, aged	Cortex	0.13	0.25	0.04	0.033	0.16				
	Lin *et al*. 2014 [128]	Glc_{16}	α-chloralose	$V_{TCA}^{g} = 0.15*(V_{TCA}^{n}+V_{TCA}^{g})$, $V_{PC} = 0.20*(V_{PC}+V_{NT})$	Fischer 344 Brown-Norway F1 rat, aged + caloric restriction	Cortex	0.21	0.39	0.07	0.053	0.26				
	Patel *et al*. 2015 [129]	Glc_{16} & Ac_2	halothane	$V_{TCA}^{g} = 0.15*(V_{TCA}^{n}+V_{TCA}^{g})$, $V_{PC} = 0.20*(V_{PC}+V_{NT})$	Wistar rat	Cortex	0.62	0.72	0.13	0.124	0.49	0.16	0.46	0.18	0.54
	Dehghani *et al*. 2016 [47]	Glc_{16}	α-Chloralose	-	Sprague Dawley rat	whole brain	0.063	0.33	0.21	0.040	0.29				
	Sonnay *et al.* 2017 [29]	Glc_{16}	α-Chloralose	-	Sprague Dawley rat, Rest	Cortex	0.070	0.53	0.31	0.064	0.45				
	Sonnay *et al.* 2017 [29]	Glc_{16}	α-Chloralose	-	Sprague Dawley rat, Somatosensory stimulation	Cortex	0.137	0.59	0.37	0.07	0.52				
	Sonnay *et al.* 2017 [117]	Glc_{16}	deep thiopental	-	Sprague Dawley rat	Cortex	0.092	0.46	0.16	0.023	0.32				
	Girault *et al*. 2019 [118]	Glc_{16}	α-Chloralose	-	Wistar rat	whole brain	0.20	0.34	0.32	0.09	0.38				
	McNair *et al*. 2022 [34]	Glc_1 & Glc_2	none; awake	several fixed relations between fluxes	Sprague Dawley rat	Cortex	0.81	1.59	0.22	0.106	0.96	0.109	0.70	0.32	1.27
	McNair *et al*. 2022 [34]	Glc_1 & Glc_2	none; awake	several fixed relations between fluxes	Sprague Dawley rat	Cerebellum	0.52	1.22	0.25	0.087	0.78	0.075	0.44	0.25	0.96
	McNair *et al*. 2022 [34]	Glc_1 & Glc_2	none; awake	several fixed relations between fluxes	Sprague Dawley rat	Hippocampus	0.80	1.18	0.19	0.114	0.74	0.079	0.72	0.30	0.88
	McNair *et al*. 2022 [34]	Glc_1 & Glc_2	none; awake	several fixed relations between fluxes	Sprague Dawley rat	Striatum	0.89	1.44	0.23	0.125	0.90	0.083	0.81	0.28	1.16
**Tree shrew**	Sonnay *et al*. 2018 [26]	Glc_{16}	isoflurane	-	tree shrew, low V_{NT}	Cortex	0.19	0.52	0.26	0.074	0.43				
	Sonnay *et al*. 2018 [26]	Glc_{16}	isoflurane	-	tree shrew, high V_{NT}	Cortex	0.23	0.58	0.32	0.066	0.49				
**Mouse**	Tiwari *et al*. 2013 [130]	Glc_{16}	urethane	several fixed relations between fluxes	C57BL6 mice	Cortex	0.46	1.15	0.25	0.02	0.71	0.10	0.36	0.24	0.91
	Tiwari *et al*. 2013 [130]	Glc_{16}	urethane	several fixed relations between fluxes	C57BL6 mice	Hippocampus	0.40	0.90	0.26	0.03	0.60	0.09	0.31	0.26	0.64
	Tiwari *et al*. 2013 [130]	Glc_{16}	urethane	several fixed relations between fluxes	C57BL6 mice	Striatum	0.29	1.02	0.19	0.06	0.64	0.11	0.18	0.27	0.75
	Tiwari *et al*. 2013 [130]	Glc_{16}	urethane	several fixed relations between fluxes	C57BL6 mice	Thalamus+Hypothalamus	0.34	1.01	0.26	0.03	0.65	0.12	0.22	0.28	0.73
	Tiwari *et al*. 2013 [130]	Glc_{16}	urethane	several fixed relations between fluxes	C57BL6 mice	Cerebellum	0.40	1.06	0.28	0.03	0.69	0.08	0.32	0.25	0.81
	Lai *et al*. 2018 [120]	Glc_{16}	isoflurane	-	Swiss nude mouse	whole brain	0.084	0.56	0.20	0.041	0.40				

Tracers: Ac_2, [2-^{13}C] acetate; Glc_1, [1-^{13}C]glucose; Glc_2, [2-^{13}C]glucose; Glc_{16}, [1,6-$^{13}C_2$]glucose; Glc_U, [U-$^{13}C_6$]-glucose.

* V_{NT} calculated with mass balance equation $V_{gln} = V_{NT} + V_{PC}$

** For 3-compartment models, $V_{NT} = V_{NT}^{Glu} + V_{PC}^{GABA}$ and $V_{TCA}^{n} = V_{TCA}^{Glu} + V_{TCA}^{GABA}$

*** Calculated as $CMR_{glc(ox)} = (V_{TCA}^{g} + V_{TCA}^{n} + V_{PC})/2$
